# Contrasting antibody responses to intrasubtype superinfection with CRF02_AG

**DOI:** 10.1371/journal.pone.0173705

**Published:** 2017-03-13

**Authors:** Colleen R. Courtney, Luzia Mayr, Aubin J. Nanfack, Andrew N. Banin, Michael Tuen, Ruimin Pan, Xunqing Jiang, Xiang-Peng Kong, Allison R. Kirkpatrick, Daniel Bruno, Craig A. Martens, Lydia Sykora, Stephen F. Porcella, Andrew D. Redd, Thomas C. Quinn, Phillipe N. Nyambi, Ralf Dürr

**Affiliations:** 1 Department of Microbiology, New York University School of Medicine, New York, NY, United States of America; 2 Department of Pathology, New York University School of Medicine, New York, NY, United States of America; 3 Faculty of Medicine and Biomedical Sciences, University of Yaoundé, Yaoundé, Cameroon; 4 Department of Biochemistry and Molecular Pharmacology, New York University School of Medicine, New York, NY, United States of America; 5 Laboratory of Immunoregulation, National Institute of Allergy and Infectious Diseases, National Institutes of Health, Bethesda, MD, United States of America; 6 Genomics Unit, Research Technologies Branch, Rocky Mountain Laboratories, Division of Intramural Research, National Institute of Allergy and Infectious Diseases, National Institutes of Health, Hamilton, MT, United States of America; 7 Department of Pathology, Johns Hopkins School of Medicine, Baltimore, MD, United States of America; 8 Veterans Affairs New York Harbor Healthcare Systems, New York, NY, United States of America; Emory University School of Medicine, UNITED STATES

## Abstract

HIV superinfection describes the sequential infection of an individual with two or more unrelated HIV strains. Intersubtype superinfection has been shown to cause a broader and more potent heterologous neutralizing antibody response when compared to singly infected controls, yet the effects of intrasubtype superinfection remain controversial. Longitudinal samples were analyzed phylogenetically for *pol* and *env* regions using Next-Generation Sequencing and envelope cloning. The impact of CRF02_AG intrasubtype superinfection was assessed for heterologous neutralization and antibody binding responses. We compared two cases of CRF02_AG intrasubtype superinfection that revealed complete replacement of the initial virus by superinfecting CRF02_AG variants with signs of recombination. NYU6564, who became superinfected at an early time point, exhibited greater changes in antibody binding profiles and generated a more potent neutralizing antibody response post-superinfection compared to NYU6501. In contrast, superinfection occurred at a later time point in NYU6501 with strains harboring significantly longer V1V2 regions with no observable changes in neutralization patterns. Here we show that CRF02_AG intrasubtype superinfection can induce a cross-subtype neutralizing antibody response, and our data suggest timing and/or superinfecting viral envelope characteristics as contributing factors. These results highlight differential outcomes in intrasubtype superinfection and provide the first insight into cases with CRF02_AG, the fourth most prevalent HIV-1 strain worldwide.

## Introduction

HIV-1 superinfection is characterized by the sequential infection of an individual with two or more genetically unrelated HIV-1 strains and provides a unique opportunity to study the adaptive immune response to challenges with multiple antigens [1, 2]. The occurrence of superinfection (SI) implies that primary infection has limited [2, 3] or even no protective effect [4–6], as deduced from comparing incidences of primary infection and SI. In some cases of SI, impaired antibody (Ab) binding and/or neutralization responses might even predispose towards a future SI event [7–10]. However, the secondary challenge of the immune system by a SI event can boost a strong immune response, as observed for various cases of SI with a different subtype (intersubtype SI) [11, 12]. The increased breadth and potency of the heterologous neutralizing antibody (nAb) response has been attributed to the elevated antigenic stimulation with diverse strains [13, 14].

In contrast, SI within the same subtype (intrasubtype SI), which generates an inherent lower genetic diversity, creates varied results, ranging from strongly enhanced to unchanged immune responses when compared to singly infected controls [12, 15–19]. Despite varied results within intrasubtype SI, a study that included a comparison of intra (n = 11) versus intersubtype SI (n = 10) nAb potencies indicated no significant mean difference [17]. Notably, a case of intrasubtype C SI has been reported that developed a very broad and potent heterologous nAb response driven by viral escape mutants and increased viral diversity [15, 20]. Comparing cases of intrasubtype SI with contrasting Ab responses allows for the study of critical parameters for the design of vaccine immunogens that generate a strong Ab response.

Data about Ab binding responses and changes of profiles upon SI is another largely missing piece in SI research. A study of intrasubtype C SI detected low amounts of preexisting gp120 and V1V2 binding IgG combined with high amounts of gp120 binding IgA in 2 out of 3 study individuals, which may have predisposed these patients towards SI [9]. A larger study of 21 HIV-1 infected subjects, including 11 intrasubtype SI cases, aimed at mapping the nAb responses to known broad neutralizing antibody (bnAb) sites. Using a single time point post-SI, the authors found no dominating nAb response to any of the 5 known bnAb sites, i.e. the CD4 binding site, V1V2 glycan, V3 glycan, the MPER region or the gp120-gp41 interphase [17]. The authors suggested the predominance of a polyspecific nAb response in these superinfected cases. In contrast, the induction of a bnAb response in an intrasubtype C superinfected individual could be clearly delineated to the V1V2 glycan region [20]. The longitudinal analysis of Ab specificities in more cases of intrasubtype SI is highly needed. Shifts or inclusion of different epitope specificities of nAb responses after SI may be a key to more effective antigen design.

So far, intrasubtype SI studies have mainly covered subtypes B [14, 16, 18, 19, 21, 22], C [5, 15, 20], and A [10, 12]. Here we characterize two cases of CRF02_AG intrasubtype SI found in Cameroon [11, 23] using a novel Next-Generation Sequencing (NGS) method and describe the longitudinal impact on the adaptive immune response. These data are the first analysis of heterologous neutralization of CRF02_AG intrasubtype SI. The recombinant subtype CRF02_AG is the dominant circulating strain of HIV-1 in Cameroon (>65%) and has spread globally since the 1960s to become the fourth most predominant strain worldwide (8%) [24–26]. We observed two contrasting responses upon intrasubtype CRF02_AG SI, which provides important insight into the factors relevant for stimulating a nAb response in natural infection.

## Methods

### Ethical considerations

This study was performed in accordance with the guidelines of the Helsinki Declaration and was approved by the Institutional Ethical Review Board of New York University School of Medicine, New York, USA and by the National Ethical Review Board in Cameroon. Written informed consent was obtained from all the participants.

### Study subjects

Intrasubtype CRF02_AG superinfected patients, NYU6554 and NYU6501, were previously identified by a heteroduplex assay screening and conformational Sanger sequencing of the *gag* gene [11]. Criteria for superinfection are genetic distances >5% between different time points of the same subject, irrespective of the genetic locus. Further details are provided in S1 Methods.

### Viral load

Viral load was determined using the Abbott m2000 RealTime HIV-1 assay as per the manufacturer’s instructions (Abbott Molecular, Des Plaines, IL).

### *Env* cloning

Briefly, viral RNA was extracted from the plasma using the QIAamp viral RNA mini kit (Qiagen Inc, Valencia, CA). Reverse transcription and nested polymerase chain reactions (PCRs) were performed with the SuperScript One-Step or two-step RT-PCR system, Platinum Taq polymerase (Life Technologies, Carlsbad, CA) to isolate a portion of *env*, (gp120+start of gp41) HXB2 region 6225–7838 (≈1600 bp). Details about cloning into pCR4 TOPO and sequencing can be found in S1 Methods.

### Phylogenetic analysis

Neighbor Joining phylogenetic trees were created using MEGA software (Kimura 2-parameter model, 200 bootstrap replications) and FigTree [27, 28].

### Recombination analysis

Detection of recombination events was performed with phylogenetic tools, Highlighter (http://www.hiv.lanl.gov/) and SimPlot analyses [29]. For details see S1 Methods.

### Next generation sequencing using miseq

Next generation sequencing (NGS) was performed at the Genomics Unit at the Rocky Mountain Laboratories, on a region of the *pol* gene (HXB2 position 2723–3225). Briefly, viral RNA was reverse transcribed, amplified and sequenced using a MiSeq NGS platform with the NEXTERA index primer sets. The protocol was modified from a previous 454 NGS based protocol [30, 31] (Illumina Biosciences), see S1 Methods for method details.

### IgG antibody isolation from plasma

IgG isolation was performed with 500 μL of heat-inactivated plasma and 450 μL of Protein G Sepharose 4 Fast Flow (GE Healthcare Life Sciences) according to the manufacturer’s instructions and as described in Klein et al. [32], additional details found in S1 Methods.

### Production and titration of HIV-1 pseudoviruses

*Env* plasmids SV-A-MLV-*env*, HIV-1 clone BaL.26, TRO.clone 11 (SVPB12), Q23 ENV17, CRF02_AG clone 250, and ZM249M.PL1 were obtained through the NIH AIDS Reagent Program, Division of AIDS, NIAID, NIH; X2131_c1 was obtained from Dr. Michael Seaman (Duke University, NC). The *env* plasmids were co-transfected together with the backbone plasmid pSG3deltaEnv (NIH AIDS Reagent Program) into 293T/17 cells according to the standard assessments protocol [33], see S1 Methods.

### TZM-bl neutralization assay

The TZM-bl assay was carried out as described in [33]. Neutralization assays were carried out in duplicates and the experiments were repeated at least twice. Neutralization curves are shown as nonlinear regression fits calculated in GraphPad Prism. IC50 values were determined in the fitted curves for the reciprocal plasma dilutions or the IgG concentration at 50% neutralization.

### ELISA

HIV-1 gp120 and gp41 binding Abs in plasma/plasma purified IgG samples were analyzed using a selected set of antigens that react well with samples from CRF02_AG infected individuals (unpublished data Duerr lab) including: a scaffolded V1V2 protein (V1V2 ZM109-1FD6) [34], a cyclic V3 peptide (V3 ZM109) [35], gp120core JRFL [36], BG505 SOSIP [37] (S1 Methods), and an MPER gp41 peptide (NIH AIDS Reagent Program, Division of AIDS, NIAID, NIH, #11938). Plasma/IgG from a Cameroonian HIV-1^-^ uninfected individual was included as a control. A standard ELISA protocol was followed, see S1 Methods for details. For ELISAs with serially diluted IgG, nonlinear regression fits were calculated and affinities derived for concentrations at half maximal binding, EC50 (GraphPad Prism).

### Epitope analysis

Amino acid consensus sequences were generated for each time point of the patient *env* sequences (functional clones) using DNAStar (Lasergene, Madison, WI) and aligned with antigen and reference sequences. For NYU6564–(2) and–(3) two consensus sequences were generated due to the appearance of two genetically separate populations.

### Breadth-potency

Breadth and potency values for the plasma samples were calculated as described in Blish *et al*. 2008 [10], see S1 Methods.

### Statistical analysis

Statistical analysis comparing the ELISA plasma binding data before and after SI to envelope immunogens was determined using a One-way ANOVA, nonparametric test with repeated measures and multiple comparisons to the time point immediately prior to SI and the time point post-SI. Significant changes (p<0.05) are marked with an asterisk.

## Results

### Two cases of intrasubtype CRF02_AG superinfection

**Fig 1:**
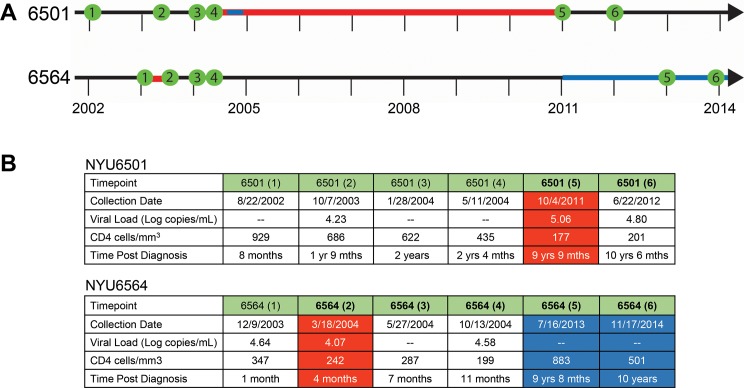
Timeline and clinical parameters of the two cases of intrasubtype CRF02_AG superinfection. **A)** Plasma samples were collected from 2002 to 2014 for patients NYU6501 and NYU6564. Samples are shown in green along the timeline. Red indicates the time span when superinfection occurred. Blue indicates antiretroviral treatment (ART). **B)** Collection dates, viral load, CD4 cell counts, and the time post diagnosis for each sample used in the study (mths abbreviates for months when listed). Red shades indicate the first time point collected after superinfection occurred. Blue shades highlight samples taken when the patient was on ART. Time points after superinfection are in bold.

Our group had previously identified two cases of intrasubtype CRF02_AG superinfection, NYU6501 and NYU6564, which both exhibited a ~6% genetic distance in *gag* (see **S1 Fig and S1 Table)** [7]. From each patient, we studied 6 plasma samples collected from 2002–2014 spanning at least 10 years including samples pre and post-SI (**Fig 1**). For NYU6501, the first sample post-SI was collected 9 years and 9 months after diagnosis and exhibited the highest viral load and lowest CD4 counts tested. SI occurred in a window of 7 years (period between last sampled time point before SI and first sampled time point when the superinfecting strain was detected) that included a period of short term antiretroviral therapy (ART) during pregnancy. Although the time between sampling was relatively large, the genetic distance between the original and superinfecting strains is greater than what would be expected from standard evolution (5%). For NYU6564, the first superinfected sample was collected only 4 months after the initial diagnosis with slightly decreasing CD4 counts and viral load. The occurrence of SI could be narrowed down to a window of 3 months without ART.

### Phylogenetic analysis of superinfection in the *env* and *pol* region

**Fig 2:**
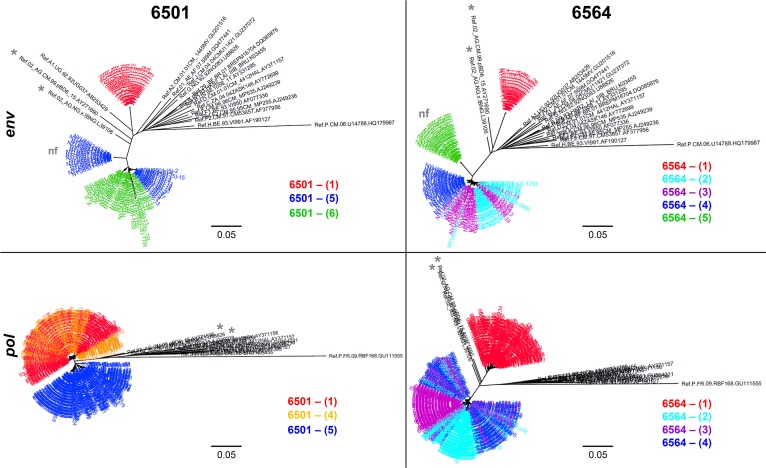
Phylogenetic diversity before and after superinfection in two genomic regions. **Top:** Envelope gene analysis for the ~1.6 kb portion (HXB2 6225–7817) for patients NYU6501 and NYU6564. Over 20 clones for at least 3 time points spanning 10 years were analyzed. Red indicates time points before SI. For NYU6501, we obtained both functional and non-functional (nf) *env* populations at the first time point post-SI (5), of which only the functional sequences evolved to closely cluster with subsequent lineages of time point 6. At the later time point NYU6564-(5), when the patient has undergone ART, we were only able to amplify non-functional (nf) *env* sequences out of the plasma. **Bottom:** The *pol* region was analyzed on the MiSeq platform generating over 40,000 sequences for each time point. Shown here are consensus sequences made from these data for ≥3 time points. Red and orange indicate time points before SI. Phylogenetic trees were generated using MEGA and FigTree software and were created using the indicated reference sequences downloaded from the Los Alamos Database (black). CRF02_AG reference sequences are marked with an asterisk.

To accurately determine the phylogenetic changes after SI, we analyzed both the highly variable gp120 region of the *env* gene using cloning and a more conserved region of the *pol* gene using a NGS platform [38] (**Fig 2**). For patient NYU6501, SI occurred between time points 4 and 5. Significant genetic distances, characteristic for SI (>5%), could be observed for *env* (17%) and *gag* (6%), but not for the conserved *pol* region (2%), highlighting the need to screen different HIV-1 genomic regions to detect superinfection (see **S1 Fig** and **S1 Table**). We observed a complete replacement of the initial strain by a new CRF02_AG variant post superinfection (**Fig 2;** see **S2A Fig**). For NYU6564 we also observed a complete shift of the initial CRF02_AG strain to a different CRF02_AG variant after SI, detected by a >20% and >5% genetic distance between time points 1 and 2 in *env* and *pol*, respectively. *Env* diversity is increased immediately after SI as evident by the appearance of two subpopulations at time points 2 and 3 (**Fig 2;** see **S2B Fig** and **S1 Table**).

### Variants post superinfection show signs of intrasubtype CRF02_AG recombination

**Fig 3:**
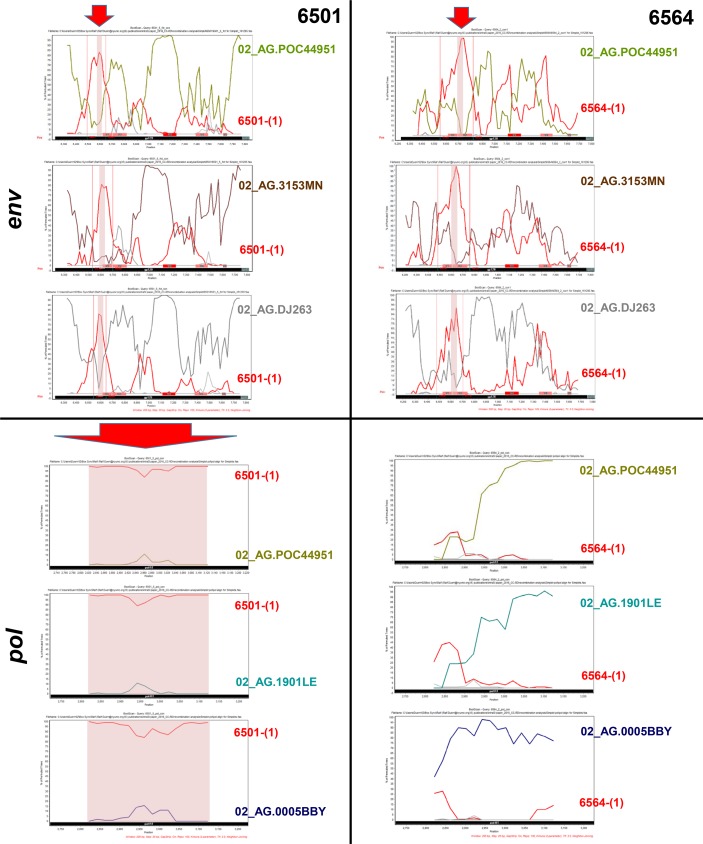
Intrasubtype CRF02_AG Recombination analysis of patient *env* and *pol* sequences with diverse CRF02_AG Reference strains. Post-SI variants from 6501 (**left**) and 6564 (**right**) were studied for signs of recombination in the *env* (**top**) and *pol* (**bottom**) region. **Top**: *Env* Simplot results of first identified *env* variants post-SI are shown for 6501-(5)fct con and 6564-(2) con1 (Query sequences), respectively. CRF02_AG Reference strains from several major 02_AG branches were studied including variants with highest similarity to post-SI patient sequences according to HIV Blast (see **S3** and **S5 Figs**); here three representative strains are included. We compared patients’ post-SI variants for recombination patterns between the representative CRF02_AG Reference strains and patient viruses prior to SI (time point 1). BootScan analyses were performed in SimPlot software with indicated strains and subtype B Reference sequences HXB2 and 1058 as outliers. The window width and step size was set to 200 bp and 20 bp, respectively. The y-axis indicates the bootstrap support; the x-axis indicates the studied *env* region. Gp120 is highlighted with a black bar, the start of gp41 with a dark green bar, and the variable gp120 regions with red bars (V3 in more intense red) at the bottom of the plot. Recurring breakpoints are indicated with vertical red lines. Recombination regions that overlay in each individual plot are highlighted with a red shadow and arrow. **Bottom**: *Pol* Simplot results of first identified *pol* sequences post-SI are shown for 6501-(5) con and 6564-(2) con (Query sequences), respectively. Recombination analyses were performed for the *pol* region, as described for *env*, between three representative CRF02_AG Reference strains and time point 1 (pre-SI) patient sequences. The studied *pol* region is highlighted with a black bar. Putative recombination regions with time point 1 variants are shown with red shadows and arrow, as observed for 6501 over the whole *pol* region.

Superinfected individuals are a common recombination source, and therefore we screened the post-SI variants for signs of recombination (**S2–S7 Figs**). In both cases post-SI sequences clustered closely with one another when analyzed phylogenetically with CRF02_AG Reference strains (**Fig 2**, **S3** and **S6 Figs**). Highlighter (**S2 Fig**) and Simplot analyses (**S4 Fig**) confirmed that at late stages post-SI no secondary recombination events occurred between the present post-SI strains and initial pre-SI variants. However, to determine if recombination had occurred between the initial and the unknown superinfecting strains to finally make up the studied post-SI strains, we analyzed patient consensus sequences at the first time point post-SI against the initial *env* infecting sequence as well as CRF02_AG reference strains (**Fig 3**, **S5** and **S7 Figs**). The related CRF02_AG Reference strains include strains that are most similar to patient post-SI variants, but for confirmation, different representatives of the major branches of CRF02_AG were also included (**S5 Fig**). Of interest, our *env* SimPlot analyses revealed recurring breakpoints in a 29 bp region in C1 for NYU6501, and a 33 bp region in V1V2 for NYU6564 that could be confirmed in multiple sequence alignments (**Fig 3 top**, **S5A–S5D Fig**). For the *pol* region, we observed no obvious signs of recombination in NYU6564, however a strong support for recombination over the whole studied region in NYU6501 (**Fig 3 bottom**, **S6** and **S7 Figs**). These results indicate minor intrasubtype CRF02_AG recombination events in NYU6564, restricted to the *env* region in our dataset, and more extensive recombination events in NYU6501, affecting both *env* and *pol*.

### Differential binding patterns to Env antigens post superinfection

**Fig 4:**
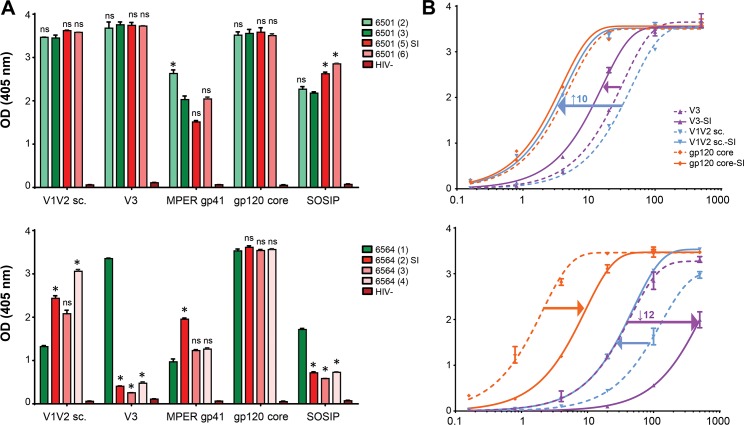
Differential binding patterns to *env* antigens after intrasubtype superinfection. **A)** Plasma samples diluted 1:100 were used in ELISA to observe longitudinal plasma antibody binding to envelope antigens V1V2 sc (scaffolded), V3, MPER gp41, gp120 core, and a SOSIP gp140 trimer. Green colors indicate samples tested pre SI and red colors indicate samples post-SI. One-way ANOVA with repeated measures and a multiple comparisons test was used to determine if the binding changes observed to the time point immediately before superinfection (dark green) were significant. **B)** Binding curves with plasma purified IgG from one time point before [6501-(3); 6564-(1)] and after SI [6501-(5); 6564-(4)] against selected antigens with >5 fold affinity change observed in at least one individual. Analyzed IgG concentrations range from 500 μg/mL to 0.1 μg/mL. Fold change in relative apparent affinities (EC50) after superinfection are indicated as arrows in the binding curves; highest fold change is indicated with the value (see also **S2 Table**). Nonlinear regression curves and EC50 values were calculated in GraphPad Prism.

To study the effects of intrasubtype CRF02_AG SI on plasma and IgG Ab binding, we performed ELISA experiments using different HIV-1 Env antigens (**Fig 4;** see **S8 Fig** and **S2 Table**). Overall, we observed much more pronounced changes in NYU6564 compared to NYU6501, evident both in plasma and IgG binding experiments.

For NYU6501, significant changes in plasma Ab binding after SI could only be observed for the SOSIP gp140 trimer. Plasma binding to V1V2, V3, and gp120 core antigens was strong and reached saturation levels at 1:100 dilution, which limited the ability to observe significant increases in binding and necessitated subsequent titration experiments. Changes in relative apparent affinities (EC50) became evident with the analysis of IgG binding curves, and we found a minor elevation in V3 (2 fold) and SOSIP (3 fold) binding, and a pronounced increase in V1V2 binding (10 fold) post-SI. For NYU6564, the strongest responses were observed against the gp120 core antigen, reaching saturation levels for all plasma samples at 1:100 dilution, but yielding a 6-fold drop in apparent affinity for IgG after SI. Plasma and IgG binding to V1V2, V3 and SOSIP antigens showed dramatic changes post-SI, weakening the initially co-dominant V3 response with a 12 fold decrease in apparent affinity. After SI, we observed a stepwise increase in plasma binding to the V1V2 antigen, which is reflected by a threefold increase in apparent affinity with IgG.

### Changes of variable loop characteristics post-superinfection

**Fig 5:**
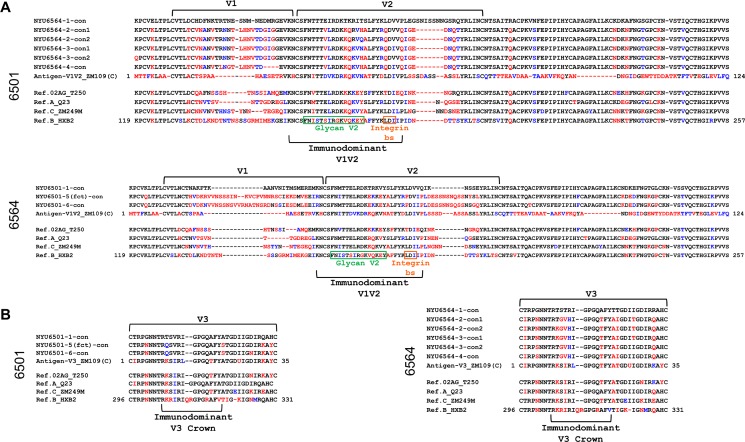
Multiple amino acid alignments of V1V2 and V3 regions with NYU6501 and NYU6564 envelope consensus sequences pre and post superinfection. Alignments were made with consensus sequences generated from all functional Env clones per time point of NYU6501 and NYU6564 (according to phylogenetic analyses in **Fig 2**); two consensus sequences per time point were created when distinct populations were detected (**Fig 2**; see **S2 Fig**). Patient Env sequences were aligned with V1V2 and V3 antigens (≥10 fold change in EC50, **Fig 4B**; **S2 Table**) and reference strains of subtypes CRF02_AG, A, C and B, also used as pseudoviruses for neutralization. Red residues indicate a nonsynonymous substitution and blue residues indicate isofunctional mutations compared to the time point 1 consensus sequence before SI, thereby indicating changes post-SI. **A)** Patient V1V2 consensus sequences aligned with the V1V2 ZM109 antigen used for binding experiments and reference sequences. Green and yellow boxes indicate the residues that make up the glycan V2 region and the integrin binding site, respectively, located within the immunodominant V1V2 region. V1 and V2 loops are indicated with brackets. **B)** Patient V3 sequences compared with the V3 ZM109 antigen and reference sequences. The V3 crown residues are denoted underneath.

Our binding experiments revealed the most pronounced changes in Ab binding (EC50 change >10) against the V1V2 and V3 antigens (**Fig 4B**). Thus, we compared the respective regions of the patients' longitudinal *env* sequences together with the V1V2 or V3 antigens in a combined amino acid alignment (**Fig 5**). Strikingly, the length of the V1V2 region increased for NYU6501 from 68 to 89 amino acids (aa) post-SI. In stark contrast, a decrease in the length of V1V2 was observed immediately post-SI for NYU6564 from 76 to 70 amino acids, mainly effecting V2, with a further decrease to 66 aa at time point 4 (**Fig 5;** see **S3 Table**). In accordance with the most pronounced changes in V1V2 binding for NYU6501 (10fold increase in apparent affinity), we observed 44 nonsynonymous changes in V1V2 post-SI, compared to 22 for NYU6564 (3fold increase in apparent affinity). For V3 binding, changes post-SI peaked for NYU6564 with a 12fold decrease in apparent affinity, reflected by 9 nonsynonymous changes in V3 (4 in the V3 crown region) and a change in predicted coreceptor tropism from X4 to R5. In comparison, there are only 4 (0 in V3 crown) nonsynonymous changes and no tropism switch (R5) for NYU6501 with modest changes in V3 binding. Critical sites known to impact V3 and V1V2 exposure (N197/A204), did not exhibit mutations post-SI [38–40]. Changes in the overall charge of the V2 glycan and V3 regions, relevant for Ab binding/neutralization and coreceptor interactions, were also observed (see **S3 Table;** Discussion) [15, 20, 34, 40].

### Variance of heterologous neutralization patterns between superinfected subjects

**Fig 6:**
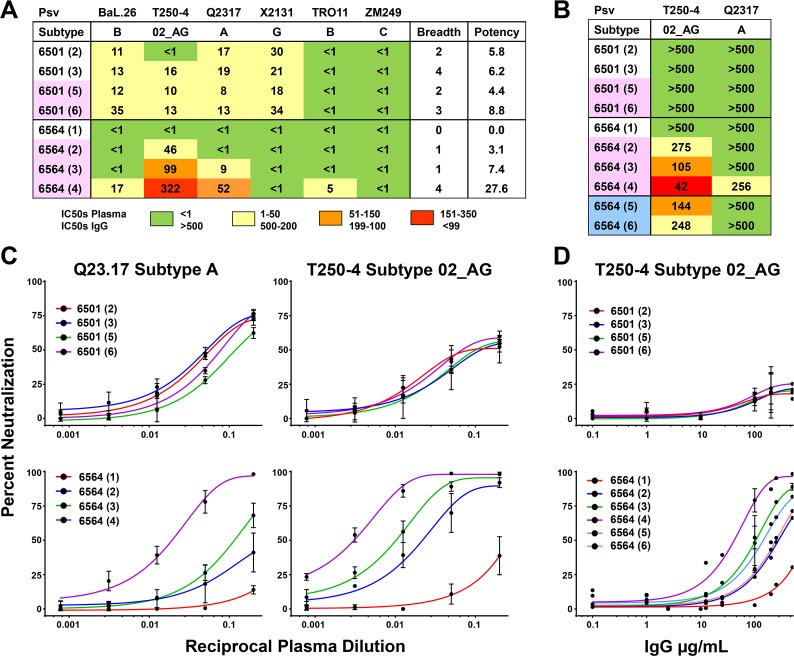
Heterologous neutralization responses in two cases of intrasubtype CRF02_AG superinfection. **A**, **B)** Table of IC50 values that represent the plasma dilutions (**A**) or IgG concentrations (**B**) needed for 50% neutralization of the respective pseudovirus. IC50 values were calculated using nonlinear regression fits of the neutralization curves in GraphPad Prism and are illustrated in a color-coded scheme. Resistance to neutralization was assumed if the plasma or IgG sample could not reach 50% neutralization at the lowest plasma dilution (<1) or highest IgG concentration (>500 μg/mL), respectively, and is indicated in the table with a green shade. Breadth and potency values were calculated as described previously [10]. Pseudoviruses are all tier 2 covering subtypes A, B, C, G, and CRF02_AG, with the exception of lab strain BaL.26, tier 1, subtype B. MLV was tested as negative control and to ensure absence of ART (**S9 Fig)**. Time points filled in with pale red are after superinfection, pale blue after initiation of ART. IC50, breadth, and potency values are calculated using the averages of 2 or more experiments. **C)** Neutralization curves from plasma samples for NYU6501 & NYU6564 against pseudoviruses T250-4 and Q23.17, shown as the percent neutralization at the reciprocal plasma dilution. **D)** Neutralization curves from IgG samples for NYU6501 and NYU6564 against pseudoviruses T250-4, shown as the percent neutralization at the given IgG concentration.

In order to determine the effects of intrasubtype CRF02_AG SI and the differential binding responses on the heterologous neutralization responses we carried out neutralization assays with both plasma and IgG to pseudoviruses (**Fig 6; S9 Fig**) and primary viral isolates (**S10 Fig**). For patient NYU6501, neutralization responses remained weak for all the longitudinal plasma samples with IC50 values not exceeding 35 (plasma dilution). Broad, yet minimally potent nAb responses were found to pseudoviruses BaL.26 (1B), T250-4 (02_AG), Q23.17 (A), and X2131 (G) as well as the virus isolate SF162 (B) without significant changes induced by SI. In contrast, NYU6564 exhibited a steady increase in neutralization after SI. The plasma sample analyzed before SI did not reach 50% neutralization for any of the pseudoviruses tested. However, post-SI we observed IC50 values reaching over 50 and 300 to tier 2 pseudoviruses Q23.17 (A) and T250-4 (02_AG), respectively, and nominal responses to subtype B pseudoviruses and primary isolates (**Fig 6; S9** and **S10 Figs**).

Analyses with purified IgG excluded unspecific effects from plasma and enabled inclusion of later time points where patients had been placed on ART (**Fig 6B and 6D**). While NYU6501 did not reach 50% neutralization for any of the pseudoviruses tested, we could confirm the increase in heterologous neutralization for NYU6564 after SI, which was equivalently found for IgG to be the highest at time point 4. Even 10 years after SI and under suppression of viral load by ART, heterologous neutralization to 02_AG was still present for NYU6564.

We further analyzed the entire gp120 Env aa sequences for changes at critical N-glycosylation sites and sites of resistance for bnAbs (**S11 Fig**). For NYU6501 we found 4 substitutions post-SI to sites known for causing resistance to CD4 binding site (CD4bs) bnAbs and a disruption of the N234 site, essential for neutralization by the gp120/gp41 interphase bnAb 8ANC195 [41, 42]. In V2, the highly variable residue 169 exhibits a Threonine (T) pre SI and Glutamic acid (E) post SI, both known to impede the development of glycan V2 bnAbs and being negatively correlated with protection in the RV144 vaccine trial [43–46]. For NYU6564, we found 4 substitutions critical for CD4bs bnAbs and in addition the substitution K169T pre SI that is replaced with K169 in viruses after SI [46]. As the site of immune pressure in RV144, the presence of a Lysine (K) at K169 was shown to be essential for the binding of protective V1V2 antibodies and ADCC, as well as driving the maturation of several broad neutralizing glycan V2 Abs. Critical N-glycosylation sites for V3 glycan, V1V2 glycan, 35O22 and 8ANC195 bnAbs remain intact during the whole course of SI.

## Discussion

We analyzed two cases of intrasubtype CRF02_AG SI that resemble each other in their phylogenetic evolution with a pattern of complete replacement with new variants post superinfection, which has been observed in several other larger SI studies [4, 5]. Both subjects further exhibit comparable genetic distances in the *gag* and *env* region between the primary and post SI strains and show signs of intrasubtype CRF02_AG recombination. However, both individuals developed highly contrasting immune responses regarding Ab binding and neutralization that necessitates deeper investigation.

Superinfection has been shown to mimic primary infection in terms of transmission and characteristics of the founder viruses [47]. In our study, this SI profile is observed with NYU6564. At the first time point post-SI, NYU6564's viral sequences exhibit short V1V2 loops (70 aa), a moderate number of potential N-Glycosylation sites in gp120 (25) and a low positive net charge in V3 (+1), characteristic for R5 tropic founder viruses in acute infection. The small region that was putatively affected by recombination has no major impact on these Env characteristics. In contrast, NYU6501's viruses at the first time point post-SI have long V1V2 loops (89 aa), a high number of potential N-Glycosylation sites (28), and a high positive net charge in V3 (+3), usually found in chronic infection [48]. It remains obscure if this pattern is directly related to the characteristics of the superinfecting strain or due to evolution during the large window when SI occurred.

While superinfection could be closely timed for NYU6564 with a window of 3 months, there is a 7 year time window in which superinfection occurred in NYU6501. Yet, the first post-SI time point, visit 5, exhibits a peak in viral load and a nadir in CD4 counts for NYU6501, indicative of a putative recent SI. Under these assumptions, NYU6501 would have experienced SI later than 9.5 years post diagnosis of primary infection, whereas NYU6564 was superinfected only a few months (1–4 months) post diagnosis of the primary infection.

The risk of SI is highest within the first year after infection, based on studies with incidence data and mathematical modeling [9, 17, 49]. An immature immune response early after HIV infection makes acutely infected patients more susceptible to SI. A boost by a genetically distinct superinfecting strain during this period may be effective at enhancing the immune response as it is known that a bnAb response usually develops within the first years of infection [50] and that early SI is a predictive factor for a stronger Ab response [17]. Despite the limitation of not having seroconversion data and the possibility that the boost in bnAb response may have occurred as lone infection too, the putatively early time point of SI might have contributed to the stronger neutralizing immune response in NYU6564, compared to the late SI in NYU6501 associated with an absent enhancement of nAbs. The occurrence of SI several years after primary infection of NYU6501 may have hit an already impaired immune system, not able to further boost the antibody response. In addition to the timing of SI, viral diversity was shown to correlate with the development of a strong immune response [13, 14]. Of interest, NYU6564 is both stimulated with a genetically more distant strain compared to NYU6501 (20% versus 17%) and also comes up with diverse *env* populations post-SI. NYU6564 time points 2 and 3 exhibit diverging populations in the *env* phylogenetic tree that corresponds to two consensus sequences in the highlighter plots, and a high within time point genetic diversity (**Fig 2**; **see S2 Fig** and **S1 Table**). For NYU6501, diversity within time points only increases very late at time point 6 which may be caused by an exhausted immune system. Since SI cannot be accurately timed in NYU6501, diversity data immediately post-SI remains obscure. It is possible that the short interval of ART during pregnancy lowered viral load and diversity, both known to drive a strong immune response [13, 14, 51]. The genetic distance analysis of NYU6501 further revealed that the new population detected after SI significantly differs in *env* and *gag*, but not in *pol* with a distance of only 2% compared to viruses pre-SI. As suggested by our recombination analysis, this might have been caused by a recombination event that occurred between primary and superinfecting strains with exclusive outgrowth of the recombinant at time points 5 and 6, which has been shown in other cases of HIV superinfection [22, 52]. Deeper insight into recombination occurring in NYU6501 and NYU6564 was impeded by the lack of data on viral populations early enough after SI to identify the full superinfecting strain prior to recombination. Our binding experiments revealed notable differences between both subjects that may have contributed to their differential neutralization profile. While NYU6501 exhibited overall higher Ab binding titers and affinities to the studied antigens in plasma and IgG ELISA experiments, NYU6564 shows much stronger changes post-SI. This suggests that a strong nAb response, as observed for NYU6564, may be better achieved by a variable polyspecific binding response post-SI rather than a higher binding response which undergoes less variation. This would also be in accordance with a recent study proposing that the nAb response in SI individuals could not be assigned to any of the known bnAb epitopes, but rather depends on polyclonal and polyspecific responses [17]. Future epitope mapping studies elaborating the nAb response in NYU6564 will give more clarification. Our epitope analysis points out that most critical bnAb epitope sites are similarly affected in NYU6501 and NYU6564. A few critical CD4bs epitopes show substitutions in both individuals post-SI. NYU6501 strains post-SI reveal a deletion of the N234 site essential for neutralization of bnAb 8ANC195 [41, 42]. The most pronounced differences were observed in Ab binding against variable regions V1V2 and V3. We could exclude known framework mutations that change the exposure of the variable loops [39, 40], thus it is likely that intrinsic features of the variable loops are decisive for the observed changes in Ab binding, and possibly for changes in neutralization. In fact, NYU6501 and NYU6564 markedly differ in these regions with significant changes post-SI. The superinfecting strains of NYU6501 exhibit very long V1V2 loops with a low net charge in the V2 glycan region (+0) that are usually found at chronic stages of the disease associated with a higher resistance to PG9 and PG16 neutralization [48, 53]. In contrast NYU6564 reveals superinfecting variants with short V1V2 regions and a high positive net charge (+3) associated with increased sensitivity to neutralization. In addition, superinfecting NYU6564 strains, harboring a Lysine at K169 replaced initial variants with the K169T substitution at the site of immune pressure known from the RV144 study. V2 Env residue 169 is one of the most variable residues in the HIV genome [43], however protective antibody functions are preferably induced and exclusively exerted upon the presence of a Lysine (K169). Mutations at K169 abrogated binding and ADCC of V2 antibodies, isolated from protected RV144 vaccinees [45, 46]. Moreover, mutations to K169 mediate viral escape for the generation of / neutralization by broadly neutralizing glycan V2 antibodies [43, 44]. While NYU6564 became stimulated with K169 carrying superinfecting quasispecies, NYU6501 exhibits variants with escape mutations both before (K169T) and after SI (K169E). It remains elusive if the putatively more immunogenic SI variants of NYU6564 with short V1V2 and K169, compared to the more immunosilent SI variants with longer V1V2 and K169E mutation in NYU6501, triggered the stronger nAb response in NYU6564.

We have provided the initial insight into intrasubtype superinfection with the most prevalent recombinant CRF02_AG and found a potent nAb response in one individual who maintained a response even after initiation of ART. The contrasting Ab binding and neutralization responses delivered valuable insight into factors that might be mandatory for successive immune stimulation. More comparative and in-depth longitudinal SI studies are needed to differentiate parameters essential for the generation of a broad and potent immune response to be applied for vaccine approaches.

## Supporting information

S1 Fig Sample timeline with genetic distances for *gag*, *pol*, and *env*. (PDF)

S2 Fig Highlighter plots reveal Env sequence diversity post SI. (PDF)

S3 Fig Phylogenetic *env* tree with diverse CRF02_AG Reference strains and patient consensus sequences. (PDF)

S4 Fig Recombination analysis of patient *env* outlier populations with pre and post SI patient consensus sequences. (PDF)

S5 Fig Recombination analysis of patient *env* post SI variants with pre SI patient variants and diverse CRF02_AG Reference strains. (PDF)

S6 Fig Phylogenetic *pol* tree with diverse CRF02_AG Reference strains and patient consensus sequences. (PDF)

S7 Fig Highlighter analysis of patient *pol* variants. (PDF)

S8 Fig Differential binding patterns of plasma purified IgG to Env antigens pre and post superinfection. (PDF)

S9 Fig Heterologous neutralization curves with pseudoviruses. (PDF)

S10 Fig Neutralization of virus isolates by NYU6501 and NYU6564. (PDF)

S11 Fig Env amino acid alignment with indicated bnAb epitope regions and sites of immune pressure. (PDF)

S1 Methods Protocols. (DOCX)

S1 Table Genetic Distances for HIV-1 genomic regions *env*, *pol*, and *gag*. (DOCX)

S2 Table EC50 values of IgG binding to Env antigens. (PDF)

S3 Table Comparison of amino acid characteristics of the envelope clones' variable loops. (PDF)
