# Supplementary material for: Contrasting antibody responses to intrasubtype superinfection with CRF02_AG
Source: PLoS One. 2017 Mar 13;12(3):e0173705. doi: 10.1371/journal.pone.0173705 (PMC5348025; doi:10.1371/journal.pone.0173705)
Supplement: S1 Fig — (PDF) [file pone.0173705.s001.pdf]

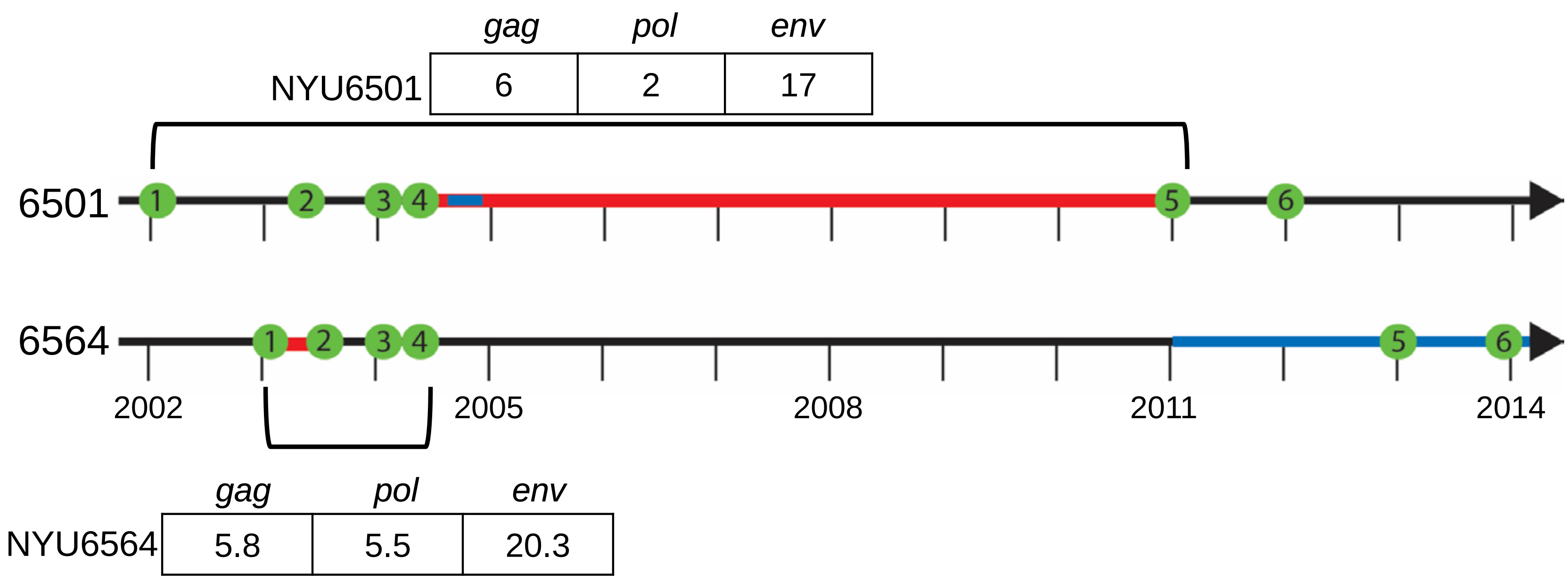

S1 Fig. Sample timeline with genetic distances for *gag*, *pol*, and *env*. Genetic distances were determined for the *gag*, *pol*, and *env* regions of both subjects between a time point before and after superinfection, and are indicated in the boxes. Additional distances were determined for *env* and *pol* spanning shorter and longer intervals (see **S1 Table**)
