# Supplementary material for: Contrasting antibody responses to intrasubtype superinfection with CRF02_AG
Source: PLoS One. 2017 Mar 13;12(3):e0173705. doi: 10.1371/journal.pone.0173705 (PMC5348025; doi:10.1371/journal.pone.0173705)
Supplement: S2 Fig — (PDF) [file pone.0173705.s002.pdf]

6501 Env highlighter

Mismatches compared to quasispecies before SI

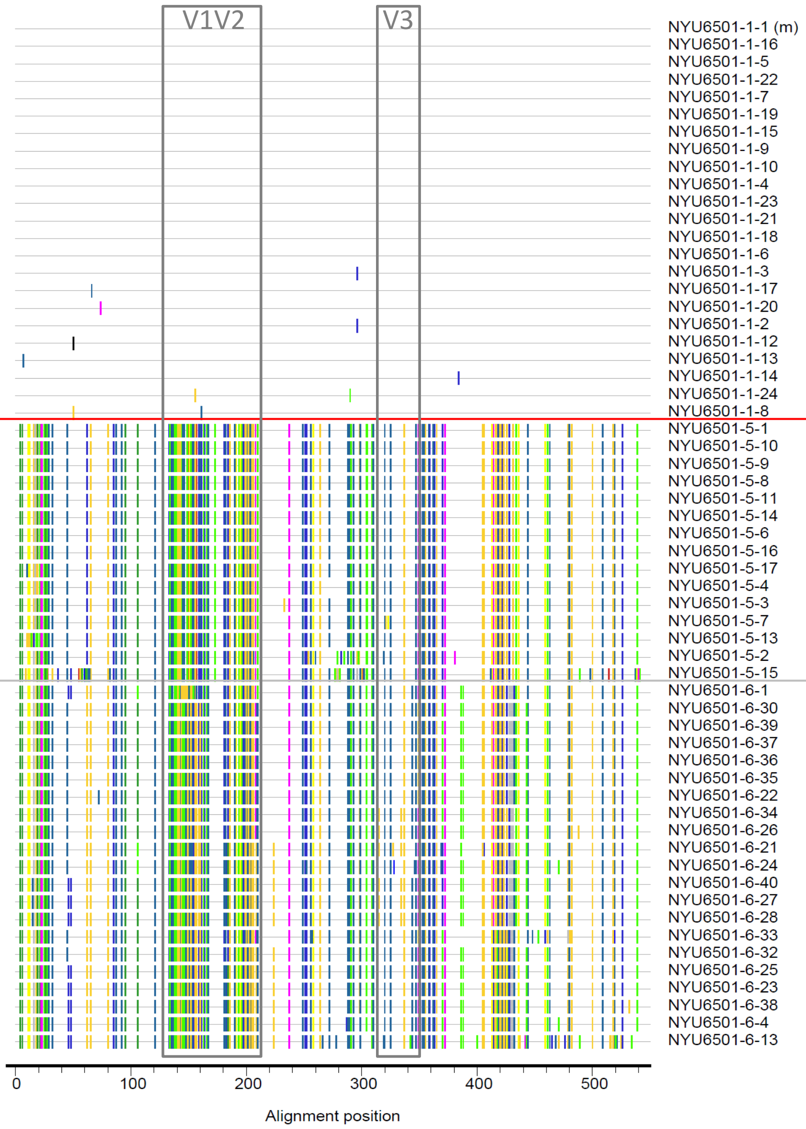

(1)

before SI

after SI

(5)

(6)

Legend

His(H)	Asp(D) Glu(E)	Lys(K) Asn(N) Gln(Q) Arg(R)
Met(M)	Ile(I) Leu(L) Val(V)	Phe(F) Trp(W) Tyr(Y)
Cys(C)	Ala(A) Gly(G) Ser(S) Thr(T)	Pro(P)
Other	Gap	

Mismatches compared to quasispecies after SI

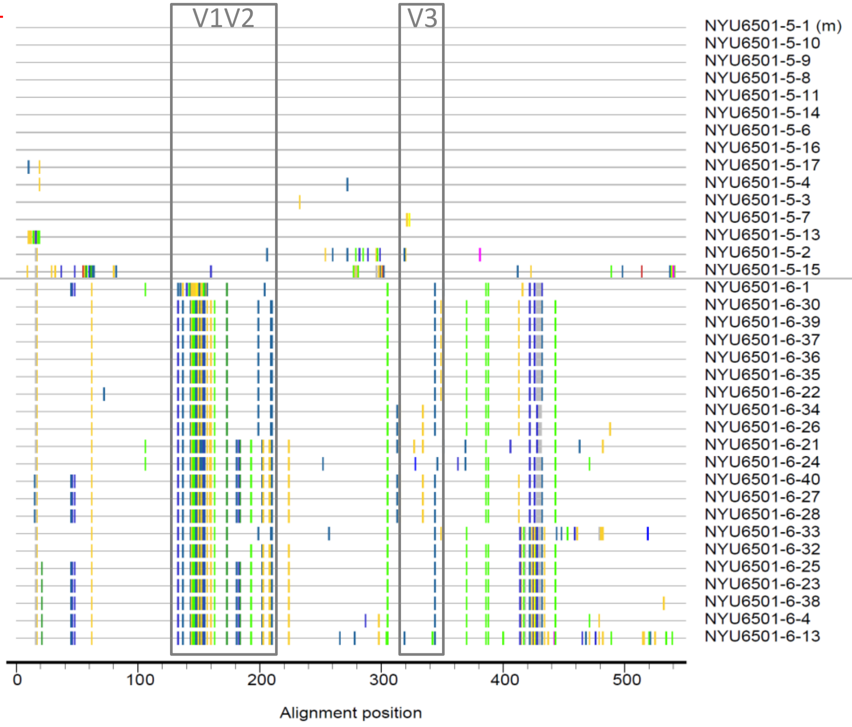

6564 Env highlighter

Mismatches compared to quasispecies before SI

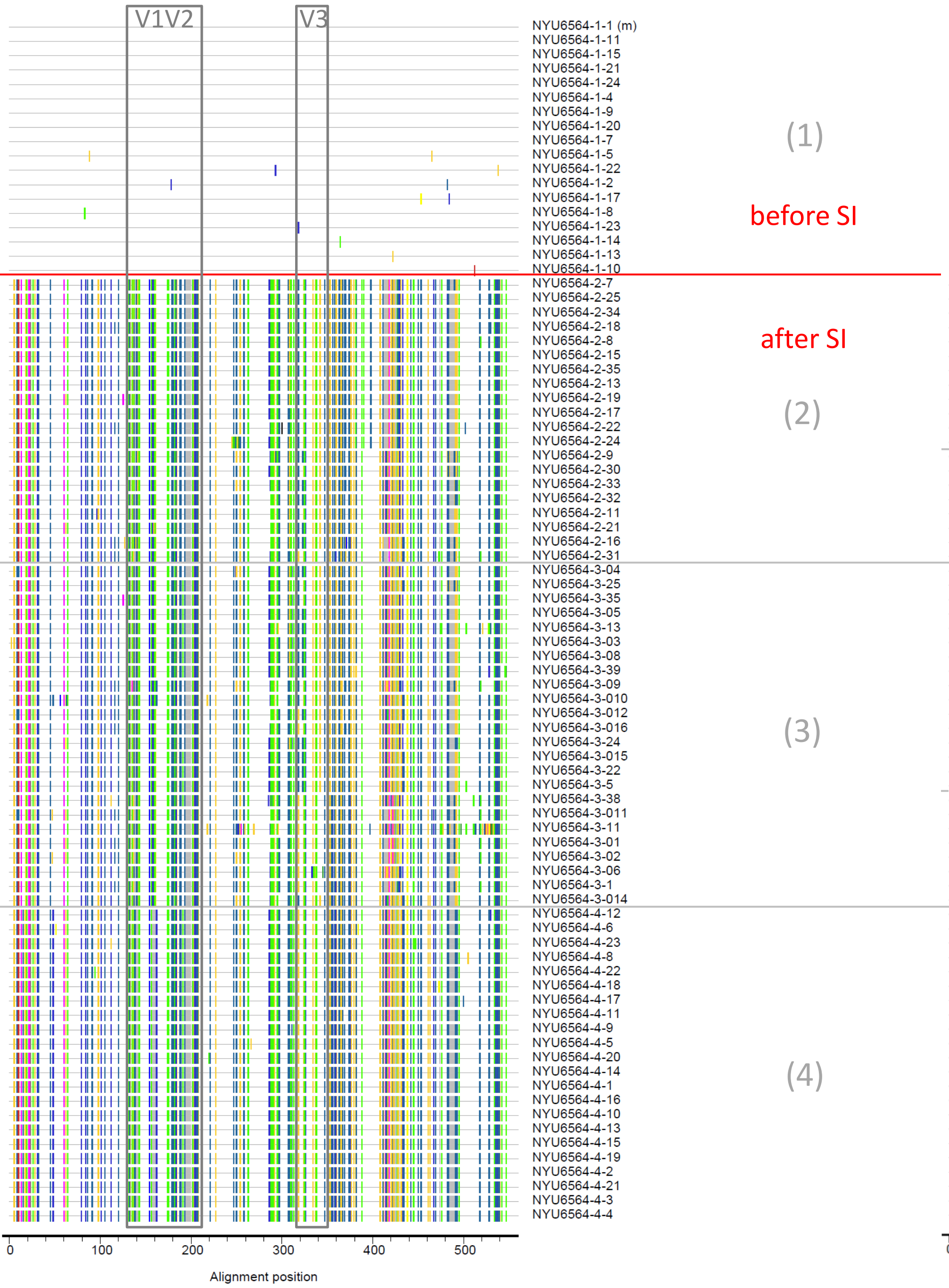

Legend

- His(H)
- Met(M)
- Cys(C)
- Other
- Asp(D) Glu(E)
- Ile(I) Leu(L) Val(V)
- Ala(A) Gly(G) Ser(S) Thr(T)
- Gap
- Lys(K) Asn(N) Gln(Q) Arg(R)
- Phe(F) Trp(W) Tyr(Y)
- Pro(P)

Mismatches compared to quasispecies after SI

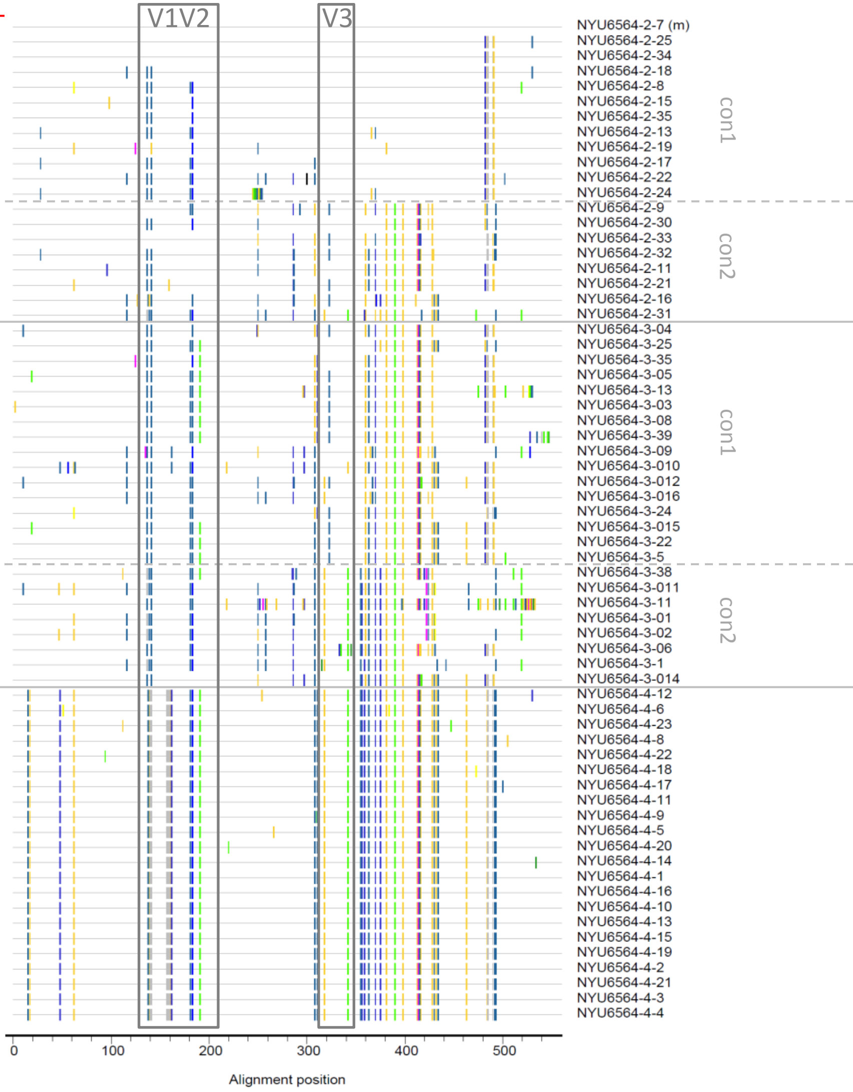

S2 Fig. Highlighter plots reveal Env sequence diversity post SI.

(A) Left: all Env clone sequences are compared to NYU6501-1-1(m) as the master. **Right:** post-SI Env clone sequences are compared to NYU6501-5-1(m) as the master sequence at the first time point after SI. (**B) Left:** all Env clone sequences are compared to NYU6564-1-1(m) as the master. **Right:** post-SI Env clone sequences are compared to NYU6564-2-7(m) as the master sequence at the first time point after SI. Highlighter plots were generated as described in methods and changes are labeled according to the color coded legend. Time points and consensus sequences (con) if applicable are indicated. V1V2 and V3 regions are shown in boxes
