# Supplementary material for: Contrasting antibody responses to intrasubtype superinfection with CRF02_AG
Source: PLoS One. 2017 Mar 13;12(3):e0173705. doi: 10.1371/journal.pone.0173705 (PMC5348025; doi:10.1371/journal.pone.0173705)
Supplement: S3 Fig — (PDF) [file pone.0173705.s003.pdf]

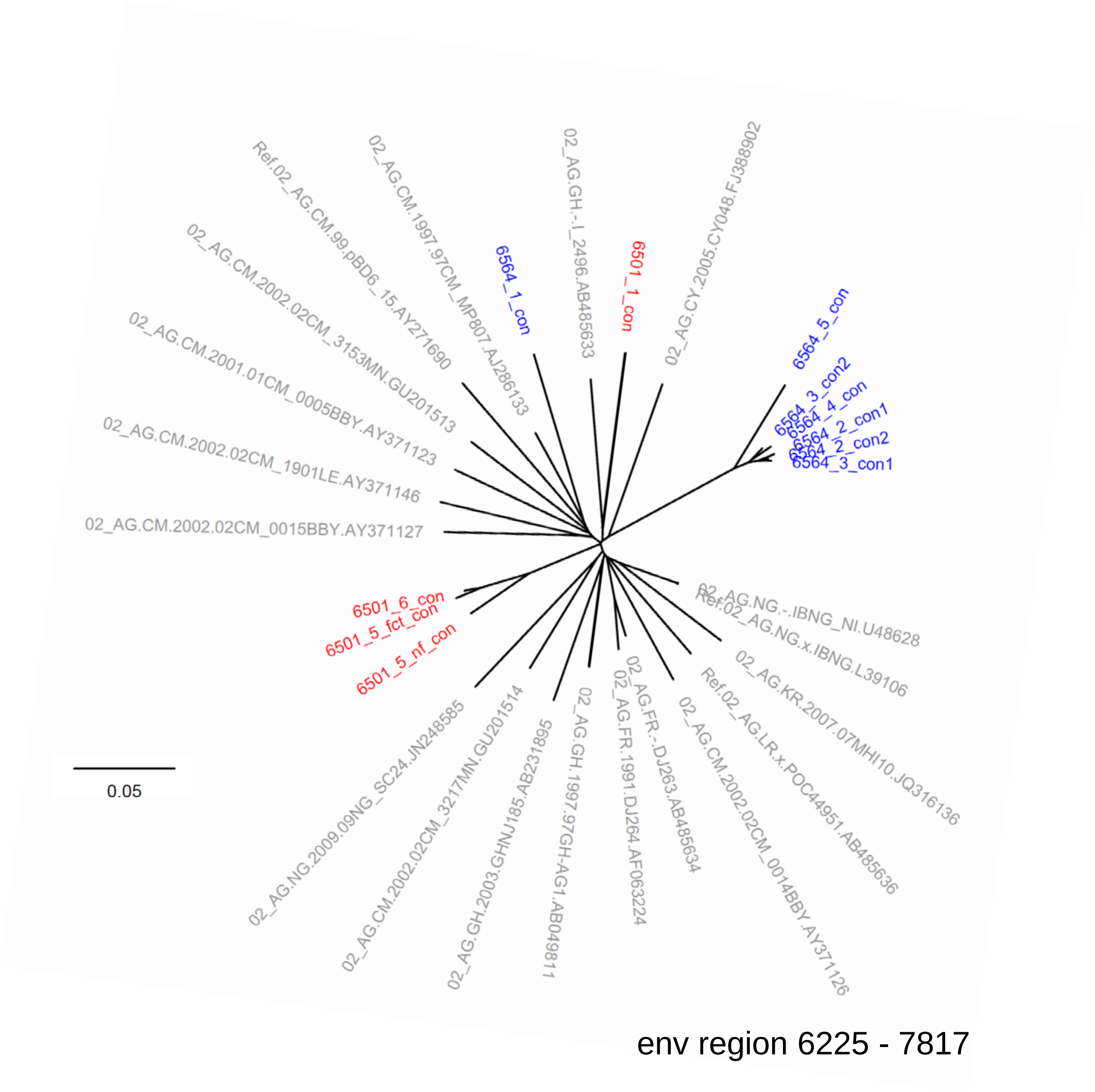

S3 Fig. Phylogenetic *env* tree with diverse CRF02_AG Reference strains and patient consensus sequences.*Envelope* consensus sequences (con) were generated for each analyzed patient time point of 6501 (red) and 6564 (blue). If two distinct populations appeared per time point, separate consensus sequences were derived (6501_5; 6564_2 and 6564_3). Patient consensus sequences were aligned with diverse CRF02_AG Reference sequences downloaded from the Los Alamos Database (grey). They include representatives of all major CRF02_AG branches and CRF02_AG strains with highest similarity to the patients' con sequences according to HIV Blast. Neighbor joining phylogenetic trees were generated using MEGA and FigTree software.
