# Supplementary material for: Contrasting antibody responses to intrasubtype superinfection with CRF02_AG
Source: PLoS One. 2017 Mar 13;12(3):e0173705. doi: 10.1371/journal.pone.0173705 (PMC5348025; doi:10.1371/journal.pone.0173705)
Supplement: S4 Fig — (PDF) [file pone.0173705.s004.pdf]

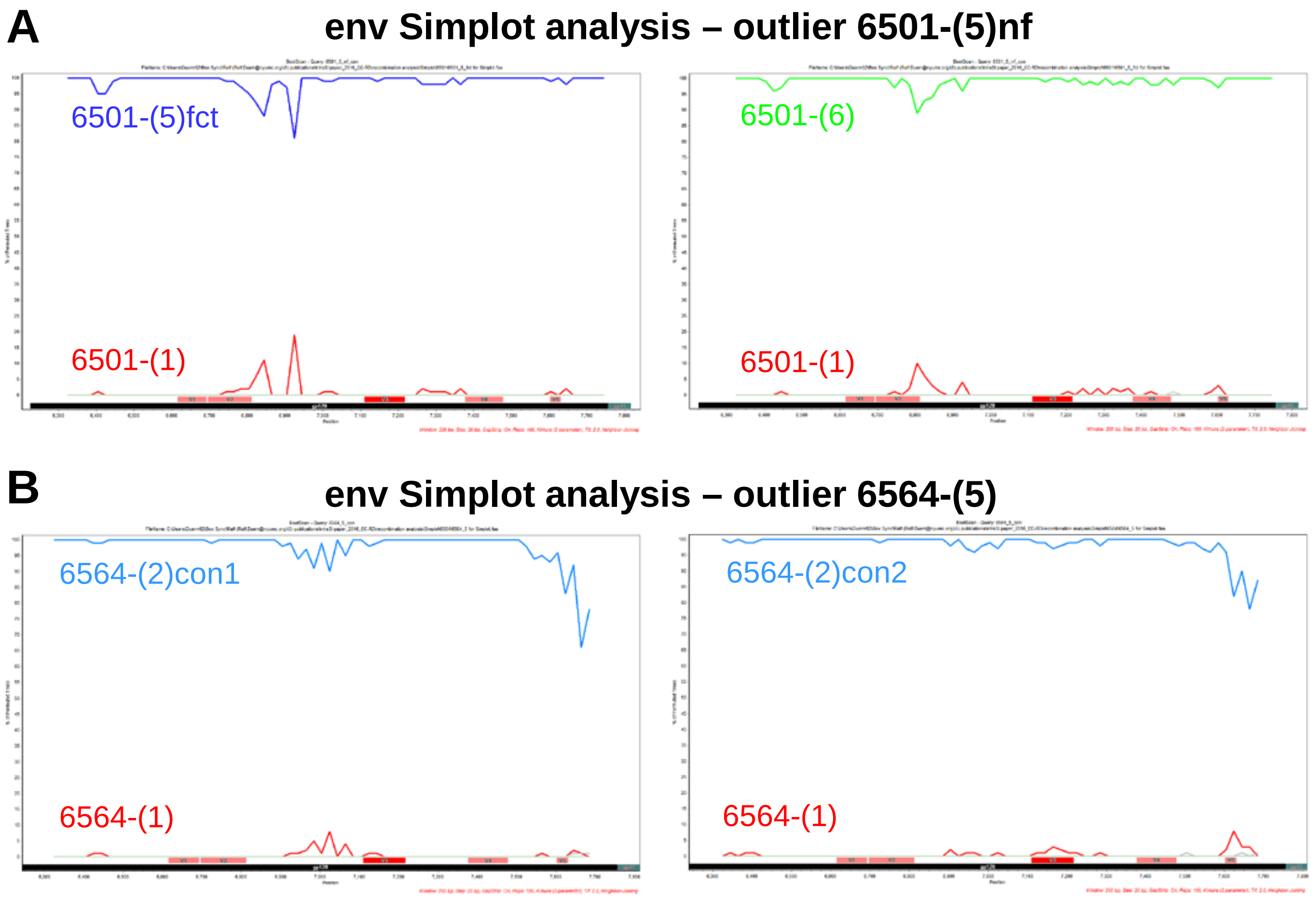

S4 Fig. Recombination analysis of patient *env* outlier populations with pre and post SI patient consensus sequences. 6501-(5)nf (**A**) and 6564-(5) (**B**) *env* sequences that appeared as outlier populations in phylogenetic trees were analyzed for recombination events between their pre and post SI variants. BootScan analyses were performed in SimPlot software with patient *env* consensus sequences. The window width and step size was set to 200 bp and 20 bp, respectively. The y-axis indicates the bootstrap support, the x-axis indicates the studied *env* region. Gp120 is highlighted with a black bar, the start of gp41 with a dark green bar, and the variable gp120 regions with red bars (V3 in more intense red) at the bottom of the plot. SimPlot analyses were performed with 6501-(5)nf con and 6564-(5) con as Query sequences in relation to the indicated patient consensus sequences and subtype B Reference sequences HXB2 and 1058 as outliers.
