# Supplementary material for: Contrasting antibody responses to intrasubtype superinfection with CRF02_AG
Source: PLoS One. 2017 Mar 13;12(3):e0173705. doi: 10.1371/journal.pone.0173705 (PMC5348025; doi:10.1371/journal.pone.0173705)
Supplement: S5 Fig — (PDF) [file pone.0173705.s005.pdf]

A

env Simplot analysis for 6501-(5)

Ref.02_Ags
vs
6501-(1)

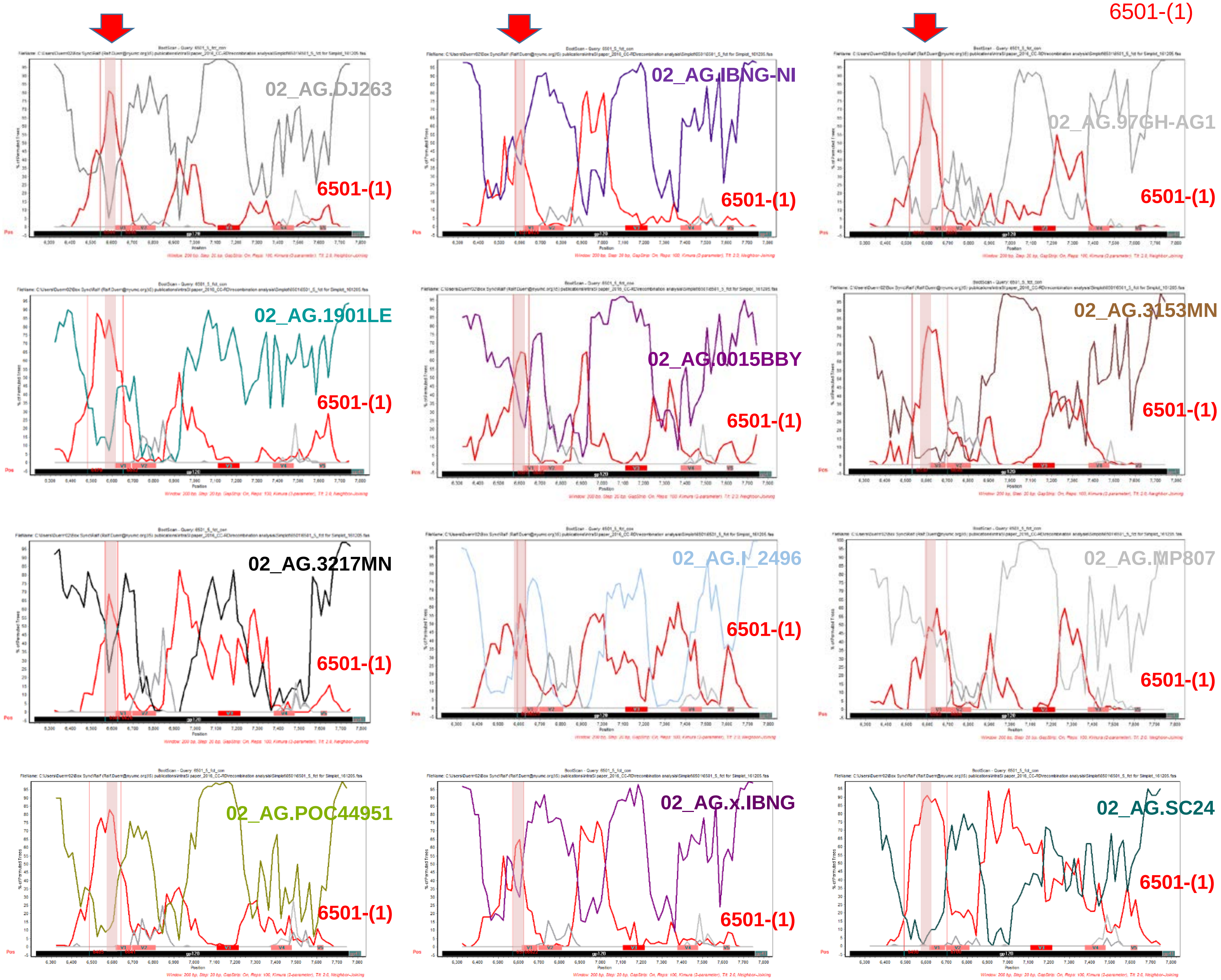

B

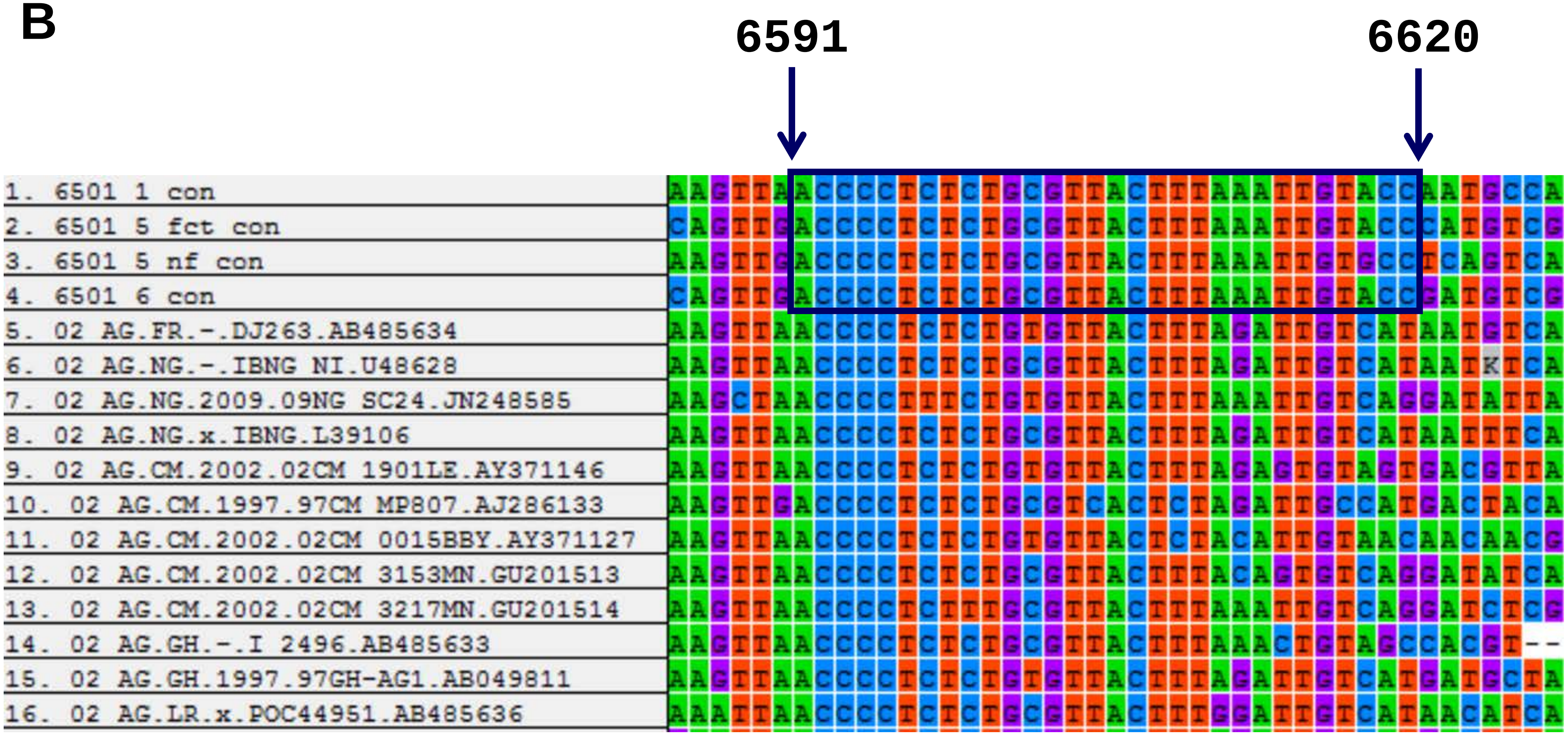
6591
6620
1. 6501 1 con
2. 6501 5 fct con
3. 6501 5 nf con
4. 6501 6 con
5. 02 AG.FR.-.DJ263.AB485634
6. 02 AG.NG.-.IBNG NI.U48628
7. 02 AG.NG.2009.09NG SC24.JN248585
8. 02 AG.NG.x.IBNG.L39106
9. 02 AG.CM.2002.02CM 1901LE.AY371146
10. 02 AG.CM.1997.97CM MP807.AJ286133
11. 02 AG.CM.2002.02CM 0015BBY.AY371127
12. 02 AG.CM.2002.02CM 3153MN.GU201513
13. 02 AG.CM.2002.02CM 3217MN.GU201514
14. 02 AG.GH.-.I 2496.AB485633
15. 02 AG.GH.1997.97GH-AG1.AB049811
16. 02 AG.LR.x.POC44951.AB485636

C

env Simplot analysis for 6564-(2)

Ref.02_Ags
vs
6564-(1)

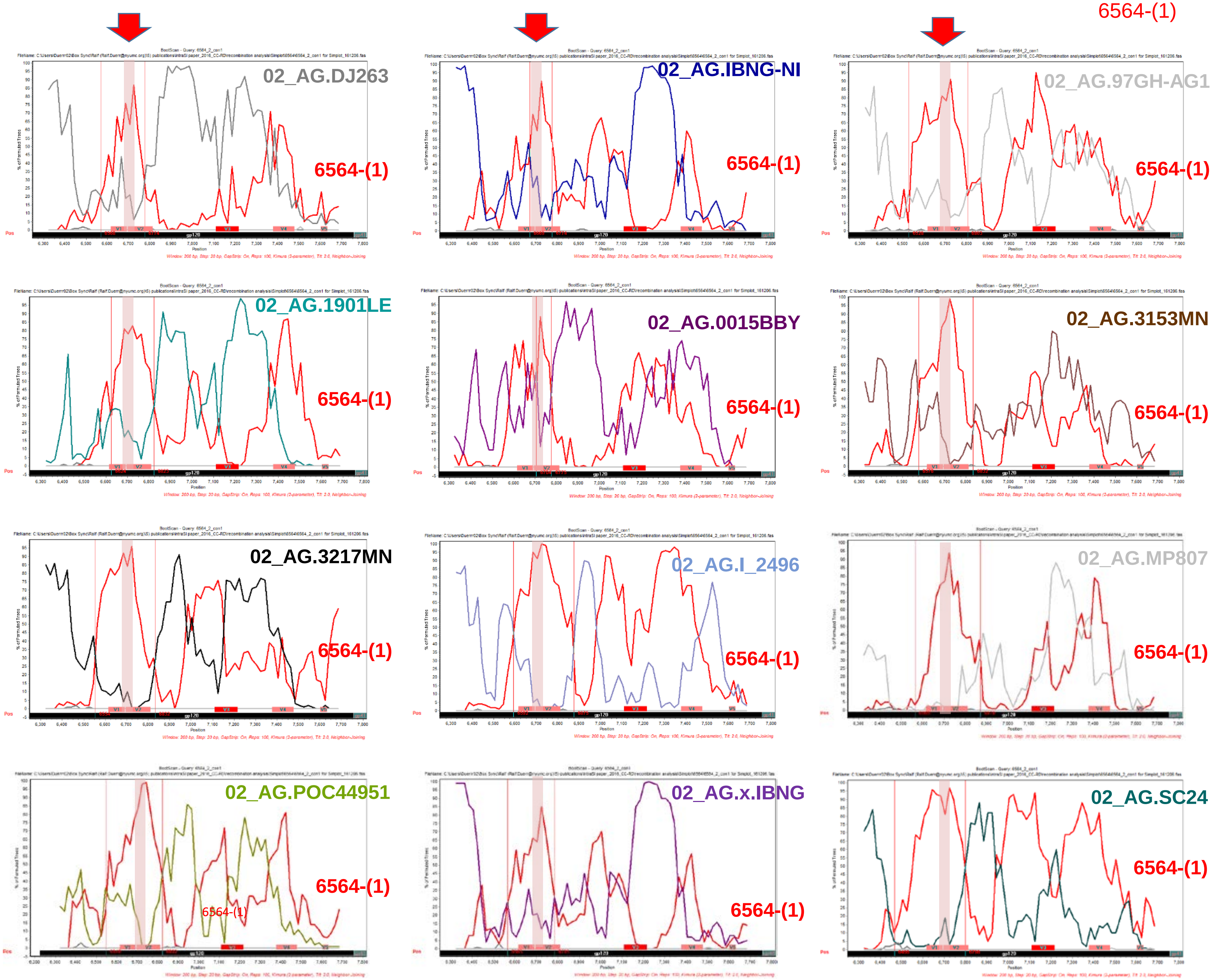

D

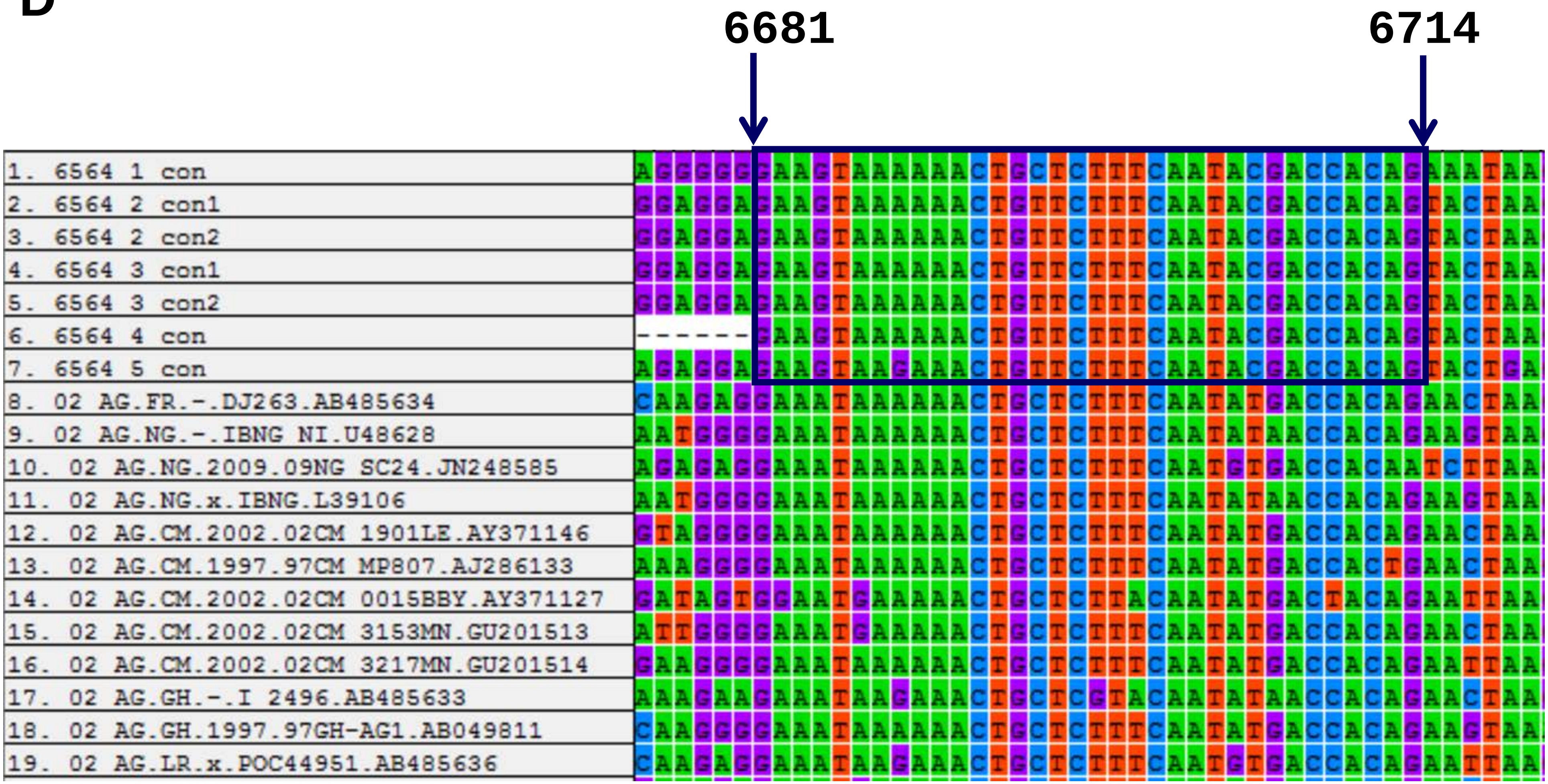
6681
6714
1. 6564 1 con
2. 6564 2 con1
3. 6564 2 con2
4. 6564 3 con1
5. 6564 3 con2
6. 6564 4 con
7. 6564 5 con
8. 02 AG.FR.-.DJ263.AB485634
9. 02 AG.NG.-.IBNG NI.U48628
10. 02 AG.NG.2009.09NG SC24.JN248585
11. 02 AG.NG.x.IBNG.L39106
12. 02 AG.CM.2002.02CM 1901LE.AY371146
13. 02 AG.CM.1997.97CM MP807.AJ286133
14. 02 AG.CM.2002.02CM 0015BBY.AY371127
15. 02 AG.CM.2002.02CM 3153MN.GU201513
16. 02 AG.CM.2002.02CM 3217MN.GU201514
17. 02 AG.GH.-.I 2496.AB485633
18. 02 AG.GH.1997.97GH-AG1.AB049811
19. 02 AG.LR.x.POC44951.AB485636

S5 Fig. Recombination analysis of patient *env* post SI variants with pre SI patient variants and diverse CRF02_AG Reference strains*.*

Post-SI *env* variants from 6501 (**A, B**) and 6564 (**C, D**) were studied for signs of recombination. **A**, **C**: Simplot results of first identified *env* variants post-SI are shown for 6501-(5)fct con and 6564-(2) con1 (Query sequences), **A** & **C** respectively. Since recombinant variants usually locate in phylogenetic trees as side branches of their closely related parental strains, we studied patients' post-SI variants for recombination patterns between related CRF02_AG Reference strains and patient viruses prior to SI (time point 1). CRF02_AG Reference strains from several major 02_AG branches were studied including variants with the highest similarity to post-SI patient sequences according to HIV Blast. BootScan analyses were performed in SimPlot software with indicated strains and subtype B Reference sequences HXB2 and 1058 as outliers. The window width and step size was set to 200 bp and 20 bp, respectively. The y-axis indicates the bootstrap support, the x-axis indicates the studied env region. Gp120 is highlighted with a black bar, the start of gp41 with a dark green bar, and the variable gp120 regions with red bars (V3 in more intense red) at the bottom of the plot. Recurring breakpoints are indicated with vertical red lines. Recombination regions that overlay in each individual plot are highlighted with a red shadow. **B**, **D**: The HIV genomic regions of putative recombination between patients' post-SI variants and time point (1) variants in 6501 (**B**) and 6564 (**D**) are indicated with arrows and boxes in DNA sequence alignments. Alignments were performed using Clustal Omega with the same patient consensus sequences and CRF02_AG Reference sequences as used for SimPlot analyses. Positions are indicated according to HXB2 numbering
