# Supplementary material for: Contrasting antibody responses to intrasubtype superinfection with CRF02_AG
Source: PLoS One. 2017 Mar 13;12(3):e0173705. doi: 10.1371/journal.pone.0173705 (PMC5348025; doi:10.1371/journal.pone.0173705)
Supplement: S7 Fig — (PDF) [file pone.0173705.s007.pdf]

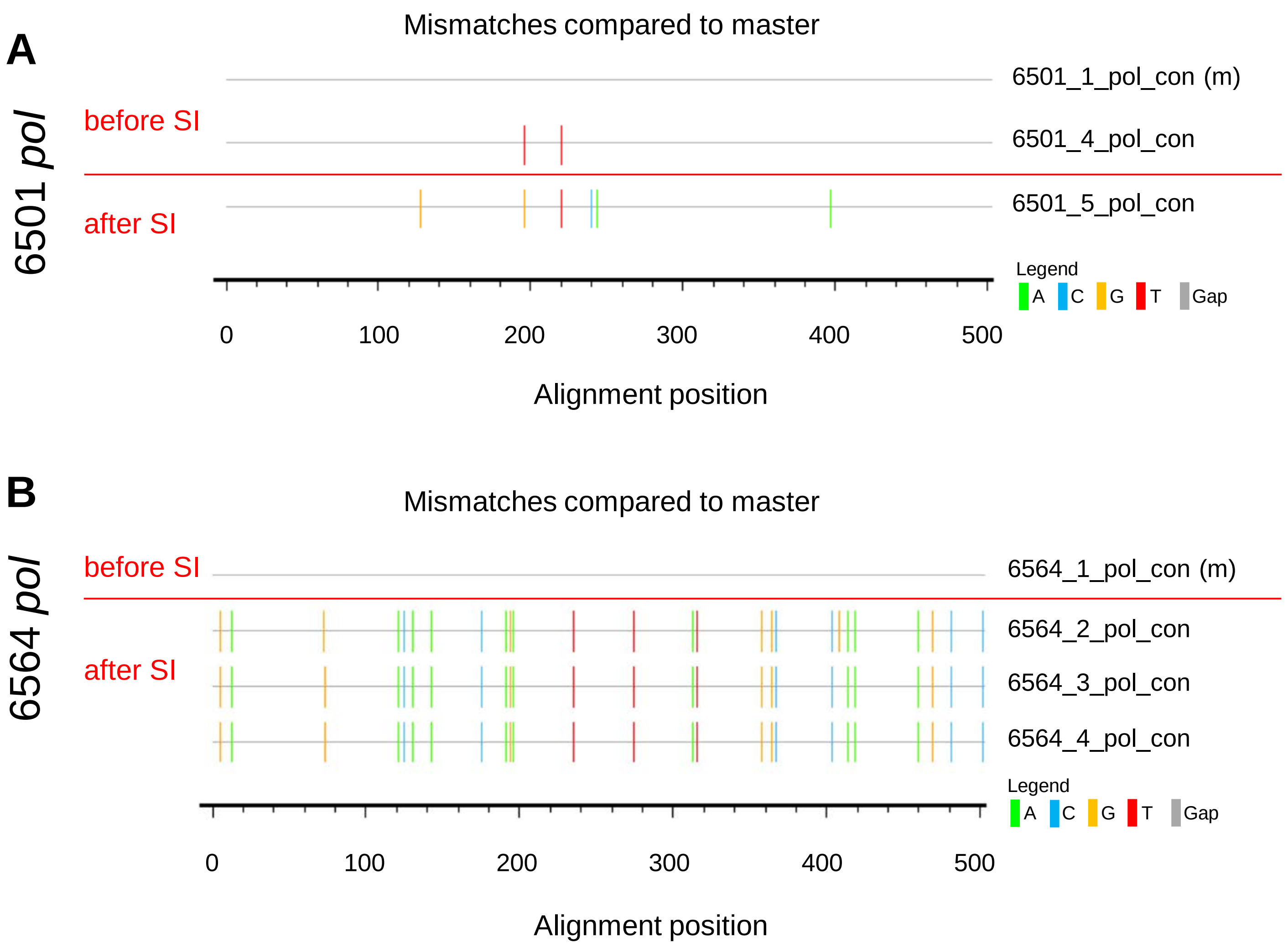

S7 Fig. Highlighter analysis of patient *pol* variants. Highlighter analyses for 6501 (**A**) and 6564 (**B**), in which *pol* consensus DNA sequences from different time points are compared to time point (1) as the master. Highlighter plots were generated as described in methods and changes are labeled according to the color coded legend. *Pol* consensus sequences (con) from time points before and after superinfection (SI) are indicated.
