# Supplementary material for: Contrasting antibody responses to intrasubtype superinfection with CRF02_AG
Source: PLoS One. 2017 Mar 13;12(3):e0173705. doi: 10.1371/journal.pone.0173705 (PMC5348025; doi:10.1371/journal.pone.0173705)
Supplement: S8 Fig — (PDF) [file pone.0173705.s008.pdf]

A

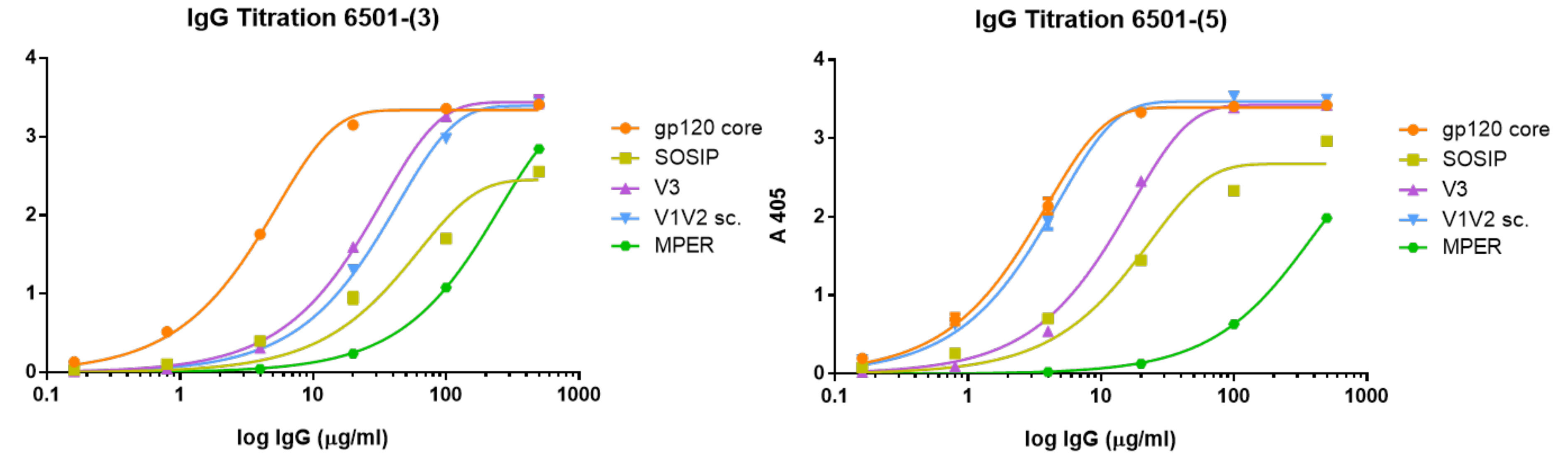

IgG Titration 6501-(3)
IgG Titration 6501-(5)
A 405
log IgG (μg/ml)
0
1
2
3
4
0.1
1
10
100
1000
gp120 core
SOSIP
V3
V1V2 sc.
MPER

B

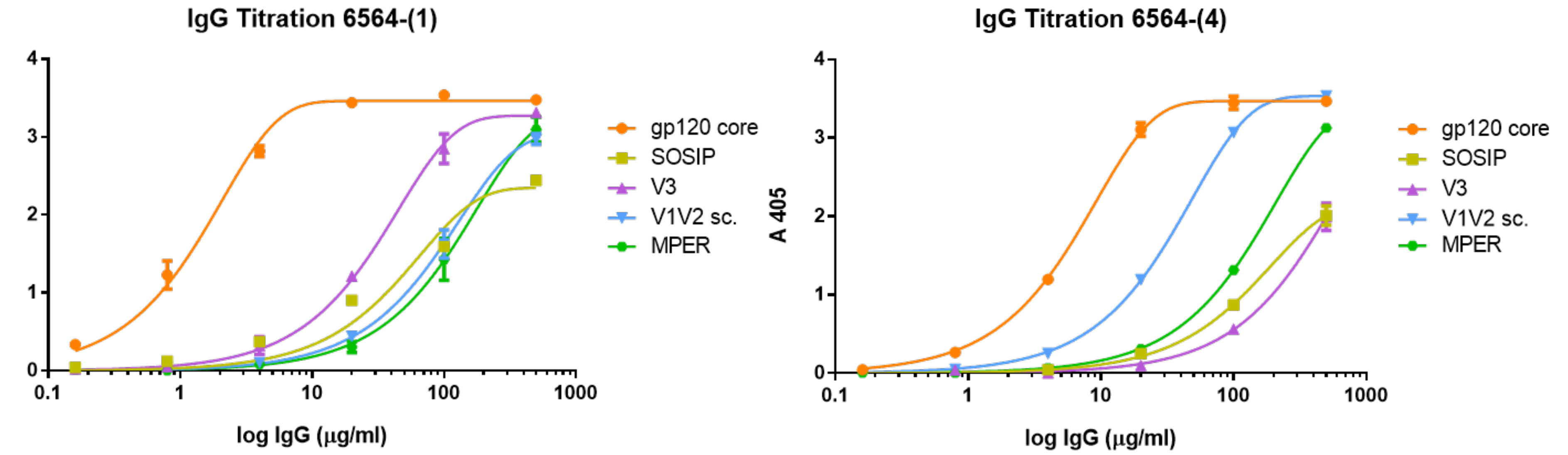

IgG Titration 6564-(1)
IgG Titration 6564-(4)
A 405
log IgG (μg/ml)
0
1
2
3
4
0.1
1
10
100
1000
gp120 core
SOSIP
V3
V1V2 sc.
MPER

C

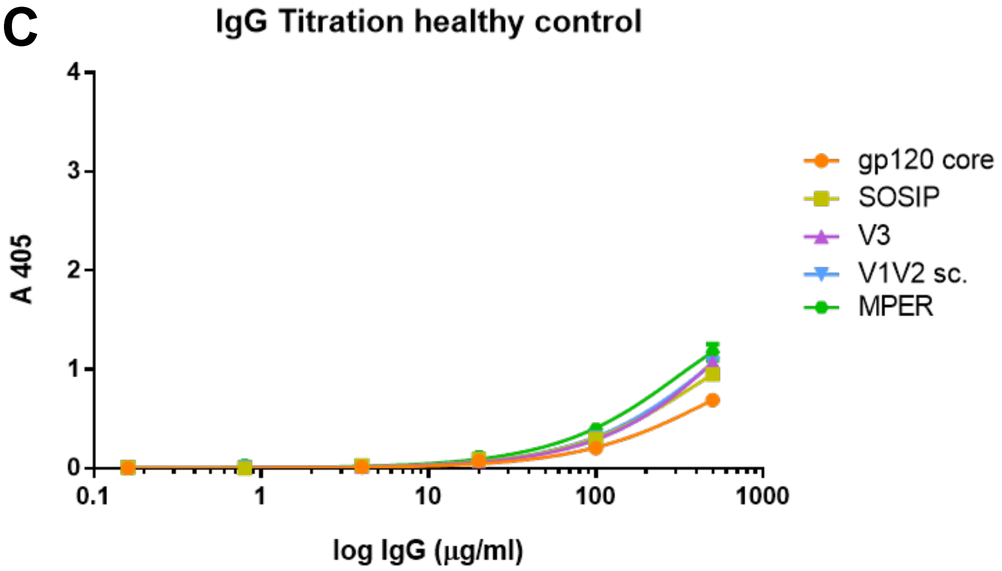

S8 Fig. Differential binding patterns of plasma purified IgG to Env antigens pre and post superinfection. **A)** ELISA binding curves of plasma purified IgG from NYU6501 time point 3, pre, and time point 5, post SI to indicated envelope antigens. **B)** ELISA binding curves of plasma purified IgG from NYU6564 time point 1, pre, and time point 4, post SI to indicated envelope antigens. **C)** ELISA binding curves of IgG purified from a healthy, HIV negative Cameroonian individual to envelope antigens. Nonlinear regression curves were fit in GraphPad Prism
