# Supplementary material for: Contrasting antibody responses to intrasubtype superinfection with CRF02_AG
Source: PLoS One. 2017 Mar 13;12(3):e0173705. doi: 10.1371/journal.pone.0173705 (PMC5348025; doi:10.1371/journal.pone.0173705)
Supplement: S9 Fig — (PDF) [file pone.0173705.s009.pdf]

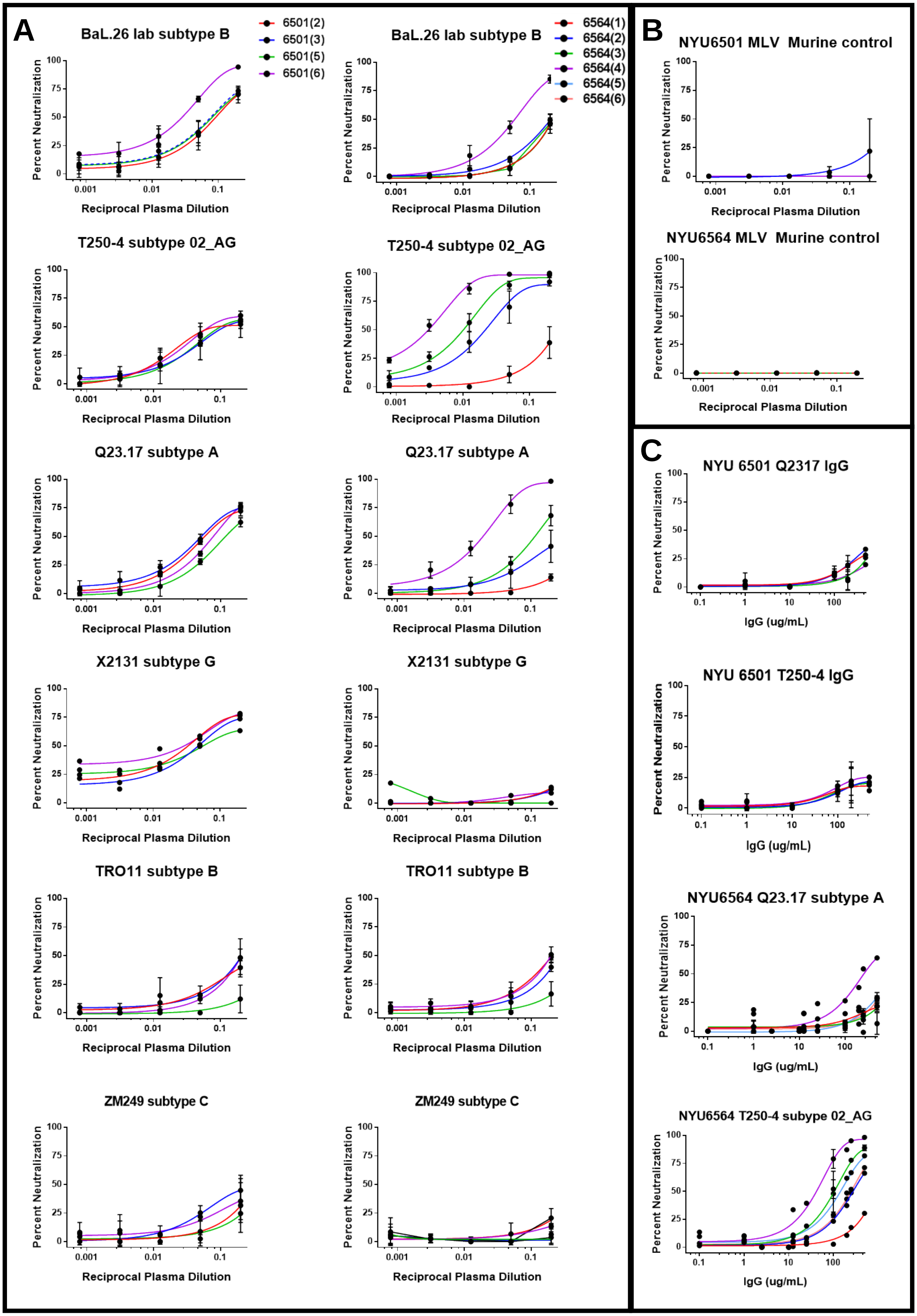
A
BaL.26 lab subtype B
6501(2)
6501(3)
6501(5)
6501(6)
Percent Neutralization
Reciprocal Plasma Dilution
BaL.26 lab subtype B
6564(1)
6564(2)
6564(3)
6564(4)
6564(5)
6564(6)
T250-4 subtype 02_AG
T250-4 subtype 02_AG
Q23.17 subtype A
Q23.17 subtype A
X2131 subtype G
X2131 subtype G
TRO11 subtype B
TRO11 subtype B
ZM249 subtype C
ZM249 subtype C
B
NYU6501 MLV Murine control
NYU6564 MLV Murine control
C
NYU 6501 Q2317 IgG
IgG (ug/mL)
NYU 6501 T250-4 IgG
NYU6564 Q23.17 subtype A
NYU6564 T250-4 subype 02_AG

S9 Fig. Heterologous neutralization curves with pseudoviruses.

A) Left: Neutralization curves for NYU6501 plasma samples, also used to calculate IC50s in **Fig 6A** main article. **Right:** Neutralization curves for NYU6564 plasma samples. **B)** MLV (Murine Leukemia Virus) neutralization curves for NYU6501 and NYU6564 plasma samples, used as negative controls. **C)** IgG neutralization curves for NYU6501 and NYU6564 to Q23.17 and T250-4, also used to calculate the IC50 values in **Fig 6B** of the main article. All neutralization assays were carried out as described in Supplementary Methods [11] and were repeated at least twice. Neutralization curves are shown as nonlinear regression fits, calculated in GraphPad Prism
