# Supplementary material for: Contrasting antibody responses to intrasubtype superinfection with CRF02_AG
Source: PLoS One. 2017 Mar 13;12(3):e0173705. doi: 10.1371/journal.pone.0173705 (PMC5348025; doi:10.1371/journal.pone.0173705)
Supplement: S10 Fig — (PDF) [file pone.0173705.s010.pdf]

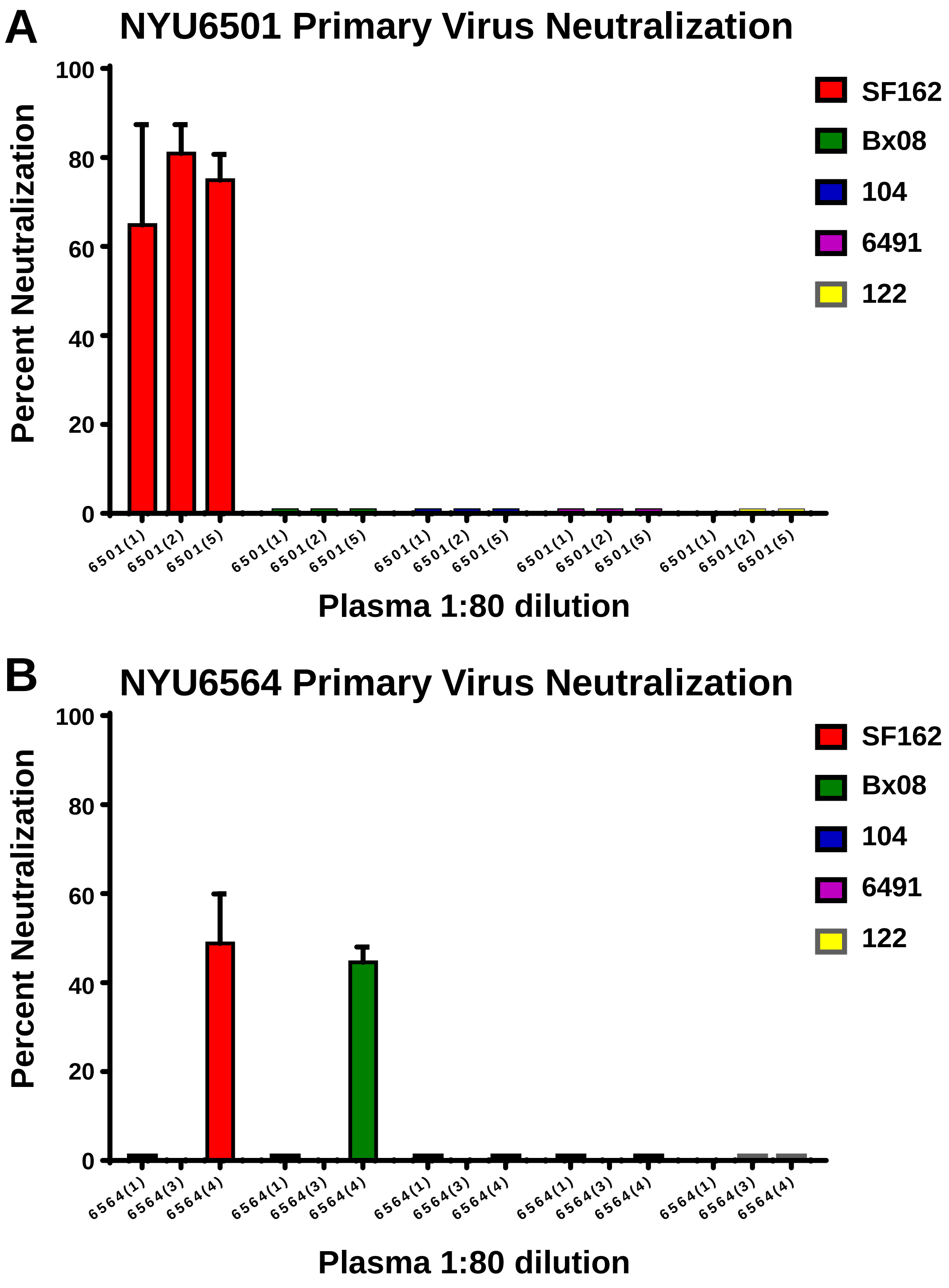

S10 Fig. Neutralization of virus isolates by NYU6501 and NYU6564. Neutralization assays carried out for **A)** NYU6501 and **B)** NYU6564 with plasma samples at 1:80 dilution to virus isolates SF162 (B) and Bx08 (B) as well as primary viruses 104 (F2), 6491 (G), and 122 (CRF02_AG). Indinavir was added to limit replication to single round infections
