# Supplementary material for: Contrasting antibody responses to intrasubtype superinfection with CRF02_AG
Source: PLoS One. 2017 Mar 13;12(3):e0173705. doi: 10.1371/journal.pone.0173705 (PMC5348025; doi:10.1371/journal.pone.0173705)
Supplement: S11 Fig — (PDF) [file pone.0173705.s011.pdf]

6564 - bnAb + CD4 epitopes / N-Glycosylation sites

```
                     1         |          |          |          |          |          |          |          |    N88   |        100
Ref.B_HXB2           MRVKEKYQHL WRWGWRWGTM LLGMLMICSA TEKLWVTVYY GVPVWKEATT TLFCASDAKA YDTEVHNVWA THACVPTDPN PQEVVLVNVT ENFNMWKNDM
NYU6564-1-con        MRVME-IQKN YPLLWRWGIM ISLLMIMCNA N-KLWVTVYY GVPVWRDAET TLFCASDAKA YDTEAHNVWA THACVPTDPN PQEMNLTNVT EKFNMWKNNM
NYU6564-2-con1       MRVMG-IQRN CPLFWRWGTI IFWIMIICSA -EQLWVTVYY GVPVWKDAET TLFCASDAKA FDTEVHNVWA THACVPTDPD PQEMELKNVT ENFNMWKNGM
NYU6564-2-con2       MRVMG-IQRN CPLFWRWGTI IFWIMIICSA -EQLWVTVYY GVPVWKDAET TLFCASDAKA FDTEVHNVWA THACVPTDPD PQEMELKNVT ENFNMWKNGM
NYU6564-3-con1       MRVMG-IQRN CPLFWRWGTI IFWIMIICSA -EQLWVTVYY GVPVWKDAET TLFCASDAKA FDTEVHNVWA THACVPTDPD PQEMELKNVT ENFNMWKNGM
NYU6564-3-con2       MRVMG-IQRN CPLFWRWGTI IFWIMIICSA -EQLWVTVYY GVPVWKDAET TLFCASDAKA FDTEVHNVWA THACVPTDPD PQEMELKNVT ENFNMWKNGM
NYU6564-4-con        MRVMG-IQRN CPLFWKWSTI IFWIMIICSA -EQLWVTVYY GVPVWKDADT TLFCASDAKA FDAEVHNVWA THACVPTDPD PQEMELKNVT ENFNMWKNGM
```

```
                                                        ---------------V1-----------|-----------------------------------V2-----------
                   101         |      R4   |          |          |   N156  N160     K169      R4       |        193
Ref.B_HXB2           VEQMHEDIIS LWDQSLKPCV KLTPLCVSLK CTDLKNDTNT NSSSGRMIME KGEIKNCSFN ISTSIRGKVQ KEYAFFYKLD IIPIDN---- ---DTTSYKL
NYU6564-1-con        VEQMHTDIIS LWEQSLKPCV ELTPLCVTLD CHDFNKTRTN -E-SNMNEDM RGEVKNCSFN TTTEIRDKTK RITSLFYKLD VVPLEGSNIS SNNGSRQYRL
NYU6564-2-con1       VDQMHQDIIS LWDQSLKPCV KLTPLCVTLT CVNANVTRNN -TLHNVTDGI GGEVKNCSFN TTTVLRDKKQ RVHALFYRQD VVQIGE---- ---DNQTYRL
NYU6564-2-con2       VDQMHQDIIS LWDQSLKPCV KLTPLCVTLT CVNANVTRNN -TLHNVTDGI GGEVKNCSFN TTTVLRDKKQ RVQALFYRQD VVQIGE---- ---DNQTYRL
NYU6564-3-con1       VDQMHQDIIS LWDQSLKPCV KLTPLCVTLT CVNANVTRNN -TLHNVTDGI GGEVKNCSFN TTTVLRDKKQ KVNALFYRQD IVQIGE---- ---DNQTYRL
NYU6564-3-con2       VDQMHQDIIS LWDQSLQPCV KLTPLCVTLT CVNANVTRNN -TLHNVTDGI GGEVKNCSFN TTTVLRDKKQ KVHALFYRQD VVQIGE---- ---DNQTYRL
NYU6564-4-con        VDQMHQDIIS LWDQSLKPCV KLTPLCVTLT CVNANVTLNG -TLHNVTD-- --EVKNCSFN TTTVLRDKKQ KVHALFYRQD IVQIGE---- ---DNQTYRL
```

```
                     ---
                   194         |          |          |   N234   |          |          |          N276  R4        293
Ref.B_HXB2           TSCNTSVITQ ACPKVSFEPI PIHYCAPAGF AILKCNNKTF NGTGPCTNVS TVQCTHGIRP VVSTQLLLNG SLAEEEVVIR SVNFTDNAKT IIVQLNTSVE
NYU6564-1-con        INCNTSAITR ACPKVNFEPI PIHYCAPAGF AILKCKDKAF NGSGPCYNVS TIQCTHGIKP VVSTQLLLNG SLAEEKVMIR TENKTDNAKI IIVQLVRPIK
NYU6564-2-con1       INCNTSAITQ ACPKVSFEPI PIHYCAPAGF AILKCNDKNF NGTGPCKNVS TVQCTHGIKP VVSTQLLLNG SLAEDEIMVR SENITNNAKI IIVQLRAPVK
NYU6564-2-con2       INCNTSAITQ ACPKVSFEPI PIHYCAPAGF AILKCNDKKF NGTGPCKNVS TVQCTHGIKP VVSTQLLLNG SLAEEKIMVR SENITNNAKI IIVQLRTPVK
NYU6564-3-con1       INCNTSAITQ ACPKVSFEPI PIHYCAPAGF AILKCNDKNF NGTGPCKNVS TVQCTHGIKP VVSTQLLLNG SLAEDEIMVR SENITNNAKI IIVQLRTPVE
NYU6564-3-con2       INCNTSAITQ ACPKVSFEPI PIHYCAPAGF AILKCNDKKF NGTGPCKNVS TVQCTHGIKP VVSTQLLLNG SLAEEEIMVR SENITNNAKI IIVQLRNPVK
NYU6564-4-con        INCNTSAITQ ACPKVSFEPI PIHYCAPAGF AILKCNDKNF NGTGPCKNVS TVQCTHGIKP VVSTQLLLNG SLAEDEIMVR SENITNNAKI IIVQLRNPVQ
```

```
                       -------------------V3-------------------                                                    -------
                   294  N301 | R4      |          |   N332   |          |          |          |          |        392
Ref.B_HXB2           INCTRPNNNT RKRIRIQRGP GRAFVTIGK- IGNMRQAHCN ISRAKWNNTL KQIASKLREQ FGNNKTIIFK QSSGGDPEIV THSFNCGGEF FYCNSTQLFN
NYU6564-1-con        INCTRPGNNT RTSTR--IGP GQAFYTTGDI IGDIRRAHCI VNKTEWDNTL QEVTTRLGEY FGINDTIIFN KSSGGDVEIT THSFNCGGEF FYCNTSRLFD
NYU6564-2-con1       IVCIRPNNNT RTGVH--IGP GQTFYAIGDI TGDIRQAHCN ISKTQWNTAL QQVGAQLQRY FGNNTTITFV NSSGGDLEVT THSFNCRGEF FYCNTSGLFN
NYU6564-2-con2       IVCIRPNNNT RKGVH--IGP GQTFYAIGDI TGDIRQAHCN ISKTQWNTTL QRVGAQLQEY FGNNTTITFA NSSGGDLEIT THSFNCGGEF FYCNTSGLFN
NYU6564-3-con1       IVCIRPNNNT RKGVH--IGP GQTFYAIGDI TGDIRQAHCN ISKTQWNTTL QRVGAQLQEY FGNNTTITFA NSSGGDLEIT THSFNCGGEF FYCNTSGLFN
NYU6564-3-con2       IVCIRPSNNT RTGVH--IGP GQTFYAIGDI IGDIRQAHCN ISKKDWNTTL QKVGAQLQEY FGNDTTITFA NSSGGDLEIT THSFNCGGEF FYCNTSGLFN
NYU6564-4-con        IVCIRPSNNT RTGVH--IGP GQTFYAIGDI IGDIRQAHCN ISKKDWNEAL QKVGAQLQEY FGNDTTITFA NSSGGDLEIT THSFNCGGEF FYCNTSGLFN
```

```
                     -------------V4----------------                                      ------V5------
                   393         |          |          |   R4     |   R4     |          |      R4  |          |      R4   483
Ref.B_HXB2           STWFNS--TW STEGSN-NTE GSDTITLPCR IKQIINMWQK VGKAMYAPPI SGQIRCSSNI TGLLLTRDGG NSN------N ESEIFRPGGG DMRDNWRSEL
NYU6564-1-con        STWRKNDNNW TNTA-NTTSN GNDTITLQCR IKQIINMWQK VGLAMYAPPI RGIIRCNSTI TGLLLTRDGG KENNSTKNGT GNETFRPGGG DMRDNWRSEL
NYU6564-2-con1       G-------TW NGTNSTI-ND SNETITLPCR IRQIVNMWQR VGQAMYAPPI SGIIRCESNI TGLLLTRDGG DNT------S SSEIFRPGGG DMRDNWRSEL
NYU6564-2-con2       GTFNG---TW NGTNSTISSD SNETITLPCR IRQIVNMWQR VGQAMYAPPI SGIIRCESNI TGLLLTRDGG DNT------N NSEIFRPGGG DMRDNWRSEL
NYU6564-3-con1       GTFNG---TW NGTNSTIS-D SNETITLPCR IRQIVNMWQR VGQAMYAPPI SGIIRCESNI TGLLLTRDGG DNT------S SSEIFRPGGG DMRDNWRSEL
NYU6564-3-con2       GTFNG---TW NGTYSNIS-N SNETITLPCR IRQIVNMWQR VGQAMYAPPI SGIIRCESNI TGLLLTRDGG NNKT----NN SNEIFRPGGG DMRDNWRSEL
NYU6564-4-con        GTFNG---TW NGTNSTIS-N STENITLPCR IRQIVNMWQR VGQAMYAPPI SGAIRCESNI TGLLLTRDGG NNT------N KKEIFRPGGG DMRDNWRSEL
```

```
                   484         |          |  gp120 ▼ gp41      |        531
Ref.B_HXB2           YKYKVVKIEP LGVAPTKAKR RVVQREKR-A VGIGALFLGF LGAAGSTMG
NYU6564-1-con        YKYKVVQIEP LGVAPTHAKR RVVEREKR-A VGLGAFFLGF LGAAGSTMG
NYU6564-2-con1       YKYKVVKIEP LGVAPTRAKR RVVEREKRAA IGLGALFLGF LGAAGSTMG
NYU6564-2-con2       YKYKVVKIEP LGVAPTRAKR RVVEREKRAA IGLGALFLGF LGAAGSTMG
NYU6564-3-con1       YKYKVVKIEP LGVAPTRAKR RVVEREKRAA IGLGALFLGF LGAAGSTMG
NYU6564-3-con2       YKYKVVKVEP LGVAPTRAKR RVVEREKRAA IGLGALFLGF LGAAGSTMG
NYU6564-4-con        YKYKVVKIEP LGVAPTRAKR RVVEREKRAA IGLGALFLGF LGAAGSTMG
```

N-Glycosylation sites of bnAbs

Site of immune pressure in RV144

Sites of resistance to CD4bs bnAbs

according to deCamp *et al.*, J Virol 2014

6501 - bnAb + CD4 epitopes / N-Glycosylation sites

```
1                                                                                        N88         100
Ref.B_HXB2          MRVKEKYQHL WRWGWRWGTM LLGMLMICSA TEKLWVTVYY GVPVWKEATT TLFCASDAKA YDTEVHNVWA THACVPTDPN PQEVVLVNVT ENFNMWKNDM
NYU6501-1-con       MRVKG-IQRN WQHLWRWGTI ILGLITICST AENLWVTVYY GVPVWRDAET TLFCASDAKA YETEMHNVWA THACVPTDPN PQEIRLENVT EEFNIWKNNM
NYU6501-5(fct)-con  MRVMG-MQRN WPPLWRGGMI IFWILMICNA -EKLWVTVYY GVPVWKDAET TLFCASDAKA YDTEAHNVWA THACVPTDPS PQEIDLKNVT ENFNMWKNNM
NYU6501-6-con       MRVMG-MQRN WPPLWRGGMI IFWILMICNA -EKLWVTVYY GVPVWKDAET TLFCASDAKA YATEAHNVWA THACVPTDPS PQEIDLKNVT ENFNMWKNNM
```

```
                                          ---------------------V1-------------------|----------------------------V2-----
101                       R4                                            N156       N160      K169       R4          187
Ref.B_HXB2          VEQMHEDIIS LWDQSLKPCV KLTPLCVSLK CTDLKNDTNT NSS------- -----SGRMI MEKGEIKNCS FNISTSIRGK VQKEYAFFYK LDIIPIDND-
NYU6501-1-con       VEQMHTDIIS LWDQSLKPCV KLTPLCVTLN CTNAKFTKA- ---------- ---ANVNITM SMEREMKNCS FNMTTELRDK TRKVYSLFYK LDVVQIK--N
NYU6501-5(fct)-con  VEQMHMDIIS LWDQSLKPCV QLTPLCVTLN CTHVDKRVVN SSSIN--KVC PVNNRSCIEK DMVEEIRNCS FNITTELRDK EKKEYALFYR PDVIPLDESS
NYU6501-6-con       VEQMHMDIIS LWDQSLKPCV QLTPLCVTLN CTDVDKNVVN SSNSVVRNAT NSRDNSSIES DMLEEIRNCS FNMTTELRDK EKKEYALFYR PDIIPLDESS
```

```
                    -----------------
188                       R4                             N234                               N276   R4   279
Ref.B_HXB2          -------TTS YKLTSCNTSV ITQACPKVSF EPIPIHYCAP AGFAILKCNN KTFNGTGPCT N-VSTVQCTH GIRPVVSTQL LLNGSLAEEE VVIRSVNFTD
NYU6501-1-con       -------SSE YRLINCNTSA ITQACPKVSF EPIPIHYCAP AGFAILKCND KEFNGTGLCK N-VSSVQCTH GIRPVVSTQL LLNGSLAETE IKIKSENISD
NYU6501-5(fct)-con  NSNQSSNYSL YRLINCNTSA ITQACPKVSF EPIPIHFCAP AGFAILKCKD EDFNGKGPCK N-VSTVQCTH GIKPVVSTQL LLNGSLAEKK IVIRSENITN
NYU6501-6-con       NNSQSSNSGQ YRLINCNTSA ITQTCPKVSF EPIPIHFCAP AGFAILKCKD EDFNGKGPCK N-VSTVQCTH GIKPVVSTQL LLNGSLAEKK IVIRSENITN
```

```
                                ----------------------V3--------------------
280                      N301 R4                        N332                                                    378
Ref.B_HXB2          NAKTIIVQLN TSVEINCTRP NNNTRKRIRI QRGPGRAFVT IG-KIGNMRQ AHCNISRAKW NNTLKQIASK LREQFGNNKT IIFKQSSGGD PEIVTHSFNC
NYU6501-1-con       NAKTLIVQLT TPVKINCTRP GNNTRTSVRI --GPGQAFYA TGDIIGDIRQ AHCNVSRSEW KETLQKVVKQ LRTHWN--KT IIFTNSSGGD LEITTHSFNC
NYU6501-5(fct)-con  NAKTILVQLI KPVRINCTRP NNNTRQSVRI --GPGQAFYS TGDIIGDIRK AYCEVNKTEW NDTLREVAKQ LREHFN--KT IIFTNSSGGD LEITTHSFNC
NYU6501-6-con       NAKTIIVQLI KPVRINCTRP NNNTRQSVRI --GPGQAFYS TGDIIGNIRK AYCEVNKTEW NDTLREVAKQ LRVHFN--KT IIFTNSSGGD VEVTTHSFNC
```

```
                          -------------------V4---------------------                                    ---V5---
379                                                         R4          R4                   R4          R4   475
Ref.B_HXB2          GGEFFYCNST QLFNSTWFN- STWS--TEGS NNTEGSDTIT LPCRIKQIIN MWQKVGKAMY APPISGQIRC SSNITGLLLT RDGGNSNNES EIFRPGGGDM
NYU6501-1-con       GGEFFYCNTA GLFNSTWNKN DSM-----ES NDTKSNDTIT LQCRIKQIIK MWQRVGQAMY APPIKGVIRC ESNITGLLLT RDGGN-NNTN ETFRPGGGDM
NYU6501-5(fct)-con  GGEFFYCNTS ALFNSTWPFN STENS-TESS NGTESNSTII LPCRIKQIIR MWQRVGQAMY APPIPGIIKC ESNITGLLLT RDGGANNGTN ETFRPGGGDM
NYU6501-6-con       GGEFFYCNTS ALFNSTWSFN STENS-TDSS NE----TNII LPCRIKQIVR MWQRVGQAMY APPIPGIIKC ESNITGLLLT RDGGANNGTN ETFRPGGGDM
```

```
476                                         |gp120 ▼ gp41              531
Ref.B_HXB2          RDNWRSELYK YKVVKIEPLG VAPTKAKRRV VQREKRAVGI GALFLGFLGA AGSTMG
NYU6501-1-con       RDNWRNELYK YKVVQIEPLG VAPTPAKRRV VQREKRAVGL GAVFIGFLGA AGSTMG
NYU6501-5(fct)-con  RDNWRSELYK YKVVKIEPLG VAPSRAKRRV VEREKRAVGL GAVFLGFLGA AGSTMG
NYU6501-6-con       RDNWRSELYK YKVVKIEPLG VAPSRAKRRV VEREKRAVGL GAVFLGFLGA AGSTMG
```

N-Glycosylation sites of bnAbs

Site of immune pressure in RV144

Sites of resistance to CD4bs bnAbs

according to deCamp *et al.*, J Virol 2014

S11 Fig. Env amino acid alignment with indicated bnAb epitope regions and sites of immune pressure.

Consensus Env amino acid sequences were aligned with reference sequence HXB2. The N-glycosite tool from the Los Alamos HIV database was used to highlight N-glycosylation sites, red. N-glycosylation sites critical for selected bnAbs are boxed in red: N88 (gp120/gp41 interphase bnAb 35O22), N156 and N160 (V2 glycan bnAbs, e.g.PG9/PG16), N234 and N276 (gp120/gp41 interphase bnAb 8ANC195), N301 and N332/N334 (V3 glycan bnAbs, e.g. PGT121/PGT128). Mutated N-glycosylation sites are highlighted in yellow. The site of immune pressure (169 in V2), as described in the RV144 vaccine trial, and sites of resistance to CD4bs bnAbs are boxed in green and blue, respectively, according to deCamp et al. 2014 [18]. Critical mutations at both sites are highlighted in respective colors. **A)** Alignment of NYU6501 Env amino acid sequences. **B)** Alignment of NYU6564 Env amino acid sequences.
