# Supplementary material for: Contrasting antibody responses to intrasubtype superinfection with CRF02_AG
Source: PLoS One. 2017 Mar 13;12(3):e0173705. doi: 10.1371/journal.pone.0173705 (PMC5348025; doi:10.1371/journal.pone.0173705)
Supplement: S1 Methods — (DOCX) [file pone.0173705.s012.docx]

**Supplemental Methods**

**Protocols**

**Study subjects**

The HIV^+^, initially drug-naïve patients NYU6501 and NYU6564 were referred to the Medical Diagnostic Center (MDC) in Yaoundé, Cameroon after a positive HIV screen result and/or for free medical consultation and CD4 count monitoring. The volunteers donated blood at the MDC from 2002 to 2014. Whole blood was shipped from Yaoundé to New York University School of Medicine, New York, NY where plasma and PBMCs were separated using Ficoll gradient centrifugation and stored at -80°C.

**Viral Load**

Viral load was determined using the Abbott m2000 RealTime HIV-1 assay as per the manufacturer’s instructions (Abbott Molecular, Des Plaines, IL), which has been demonstrated to accurately access CRF02_AG samples. Briefly, the m2000 RealTime HIV-1 assay performs automated extraction (input volume of 0.6 ml, m2000sp apparatus), a real-time PCR amplification of the integrase gene fragment, and a noncompetitive fluorescent detection (m2000rt instrument, dynamic range of 40 to 10^7^ copies/ml) [[1](#_ENREF_1)].

**Incidence testing**

To distinguish chronic (≥ 6 months) and acute (< 6 months) infections, a multi-assay algorithm (MAA) was used including the LAg-Avidity assay (HIV-1 LAg-Avidity EIA, SEDIA Biosciences Corporation, Portland, OR) and the BioRad-Avidity Assay based on the Genetic Systems 1/2+O ELISA (Bio-Rad Laboratories, Redmond, WA) [[2](#_ENREF_2)]. Both assays were performed according to the manufacturer’s instructions. Briefly for the Lag-Avidity assay, plasma was incubated for 60 minutes at 37°C with the HIV-1 antigen (rlDR-M). Disassociation buffer was added to remove antibodies with low avidity and then goat anti-human IgG-HRP was added to bind the bound IgG. TMB substrate was used to initiate a color change reaction read as optical density. The BioRad-Avidity Assay was carried out in the same fashion with the following modifications, the initial incubation is carried out at 4°C and diethyl amine was used as a chaotropic agent to disrupt binding of antibodies with low avidity. Results from both assays were compared with the established internal controls to calculate the duration of infection.

***Env* cloning – colony PCR and sequencing**

Products were cloned into the pCR4 TOPO cloning vector according to the manufacturer’s instructions (Life Technologies, Carlsbad, CA) and transformed into One Shot TOP10 competent *E.coli*. Screening for positively transformed *E.coli* colonies was performed by colony PCR using Phusion 2x Master Mix, universal vector specific primers M13F / R (pCR4 TOPO) or T7 / BGH rev (pcDNA3.1), and colonies diluted in 100 μl LB medium as template (0.5 μl). Positive clones were cultured in LB_Amp_ medium, and then plasmids were isolated using the QIAprep Spin Miniprep kit according to the manufacturer’s protocol (Qiagen inc, Valencia,CA). Plasmids were sequenced for the insert portion using universal primers M13F / R (pCR4 TOPO) or T7 / BGH rev (pcDNA3.1). Sequence analysis and alignment was performed using DNASTAR and Clustal W [[3](#_ENREF_3)].

**Genetic Distance**

To calculate the genetic distance within and between different time points of each patient, sequences were grouped according to time point in MEGA and analyzed with the compute mean distance analysis [[4](#_ENREF_4)].

**Highlighter Plots**

All functional Env clone sequences (translated protein sequences) were aligned using Clustal Omega and Highlighter plots were created using the Highlighter tool provided by the Los Alamos HIV sequence database (<http://www.hiv.lanl.gov/>). For the Highlighter analysis, sequences were sorted first according to time point, and second to similarity. First, Highlighter analyses were performed using a representative quasispecies of the first time point before superinfection as a master. Second, in order to study the genetic changes that evolved post SI, a representative quasispecies of the first time point after superinfection was used as a master, against which all subsequent other sequences were compared.

For the more conserved *pol* region, NGS DNA sequences were used to generate one representative DNA consensus sequence per time point. The representative consensus sequences for all analyzed time points per patient were subjected to a comparative Highlighter analysis.

**Tropism testing**

Coreceptor tropism was predicted for the V3 regions of the Env consensus sequences using the genotypic prediction tool Geno2pheno coreceptor 2.5 (http://coreceptor.geno2pheno.org/) with the false positive rate of 5%, as recommended for the prediction of CRF02_AG strains [[5](#_ENREF_5)].

**Simplot**

To identify possible recombination events and their breakpoint regions, the BootScan tool was applied from SimPlot version 3.5.1 [[6](#_ENREF_6)]. For all SimPlot analyses, the window width and step size was set to 200 bp and 20 bp, respectively. Patient consensus sequences, deduced from the *env* and *pol* regions, were analyzed for the occurrence of recombination events between pre and post SI patient sequences, or between pre SI patient and CRF02_AG Reference sequences.

**Recombination analysis**

The screening for possible recombination events was performed with the help of phylogenetic tools, Highlighter and SimPlot analyses. Highlighter plots indicate the extent of genetic changes between different sequences and different time points over the whole genomic region being studied. SimPlot analysis reveals putative recombination breakpoints between two selected sequences. SimPlot was performed to check if patient outlier populations are the result of recombination between patient derived sequences from time points before and after superinfection. In addition, initial variants post superinfection were studied for signs of recombination events which already occurred. Since recombinant viruses appear in phylogenetic trees as side branches of their closely related parental strains, we analyzed post SI variants for recombination patterns between related CRF02_AG Reference strains and patient viruses prior to SI. For a thorough analysis, we studied the CRF02_AG Reference strains from all major 02_AG branches, including variants with highest similarity to post SI patient sequences, according HIV Blast.

**MiSeq protocol**

Next generation sequencing (NGS) was performed on a region of the *pol* gene (HXB2 position 2723 - 3225) and env gp41 gene (HXB2 position 7938 - 8256). NGS data in the *pol* region was obtained for all samples studied. NGS data in the gp41 region could not be generated for all samples tested and was omitted from results, but included in the MiSeq protocol. This protocol was modified from previous method[[7](#_ENREF_7), [8](#_ENREF_8)].

**Part 1: Extraction**

**Equipment and Reagent needed that are NOT in the Kit:**

- QIAamp Viral RNA Mini Kit (catalog numbers #52904 or #52906)
- Ethanol (96-100%)
- 1.5 microcentrifuge tubes (tubes only removed by dumping small amount into a sterile hood – DO NOT reach into bag with gloved hand)
- Sterile, RNase-free 20uL, 200µL & 1000µL pipet tips (pipet tips with aerosol barriers for preventing cross-contamination)
- 15mL polystyrene conical tubes
- 3 Racks to hold 2 mL tubes (one 16x5 and two 8x12 racks)
- Vortex – kept within the sterile hood in pre-amp area
- Microcentrifuge
- RNAse Away

**Template preparation:**

| Study Name, Extraction #, Time Point if applicable, Date of Extraction (ALIVE Extraction 4 TP1 5/19/2015) | | |
| --- | --- | --- |
|  | Sample ID | Sample Location |
| 1 | Sample ID 1 (A93445) | Box Location of Sample ID 1 (A8) |
| 2 | Sample ID 2 (A93446) | Box Location of Sample ID 2 (C4) |

- Before protocol, make sure to make an extraction template to easily keep track of extracts. Template should contain each sample to be extracted, and should uniquely identify each sample with a number from 1 to n.

**Cleaning Process:**

Wear laboratory coat specific for Pre-Amp Room. Take off laboratory coat when leaving Pre-Amp Room.

1. Spray pre-amp lab bench with RNAse Away, wipe down.
2. Repeat with EtOH, wipe down.
3. Get Qiagen box from shelf and place on bench.
4. Wrap buffer in parafilm and place in water bath (56⁰C) to dissolve crystals.
5. Put pipets, racks, pen, and microcentrifuge in the hood (vortex already in hood).
6. Spray all equipment and hood surface with RNAse Away.
7. Wipe down from right to left, making sure everything including the hood surface is dry.
8. Spray again but with 70% Ethanol and wipe down the same way.

**Preparation of Reagents:**

EtOH:

1. On clean pre-amp lab bench, aliquot 560uL x (n+1) EtOH into a 15mL conical tube (n = # of extractions).

- **14 mL** for standard plate (24 samples).

1. Bring to hood.

AW1 and AW2:

1. For first time use of kit: On clean lab bench add appropriate amount (indicated on bottles) of 96-100% ethanol to AW1 and AW2 bottles using a serological pipet.

- Store at room temperature – stable until kit expiration date.

1. Pipet 0.5mL * (n+1) of AW1 and AW2 into 2 separate 15 mL conical tubes, labeled AW1 and AW2 respectively. **12.5** **mL** for standard plate.
2. Bring both 15 mL conical tubes to hood.

Preparation of AVL Buffer-Carrier RNA solution:

*Note: Buffer AVL-carrier RNA should be prepared fresh and is stable at 2-8°C for 48 hours.

1. If available, thaw carrier RNA. Otherwise, reconstitute the carrier RNA by adding 310 µL Buffer AVE to the tube containing 310 µg lyophilized carrier RNA (red tube top).
   1. Dissolve thoroughly by vortex and store at -20°C
   2. Do not freeze-thaw each aliquot more than three times
      1. Throw out any remaining volume after the third thaw
2. Remove buffer AVL from water bath and check for precipitate. If precipitate crystals remain, incubate at 56°C until completely dissolved.
3. Look up the volume of Buffer AVL-carrier RNA mix needed per batch of samples by selecting the number of the samples to be processed in chart below.
4. Add mL amount of AVL buffer to a 15 mL polystyrene conical tube. Place both AVL bottle and 15mL tube in hood.
5. In hood, add the uL quantity of AVL and the corresponding quantity of carrier RNA.
6. Mix by inversion. **Do NOT vortex**
7. Set aside.

**14mL** Buffer AVL + **140 uL** RNA-AVE for standard plate

| **No. of Samples** | **Vol. Buffer AVL (mL)** | **Vol. of Carrier RNA-AVE (µl)** | **No. of Samples** | **Vol. Buffer AVL (mL)** | **Vol. of Carrier RNA-AVE (µl)** |
| --- | --- | --- | --- | --- | --- |
| **1** | 0.56 | 5.6 | **13** | 7.28 | 72.8 |
| **2** | 1.12 | 11.2 | **14** | 7.84 | 78.4 |
| **3** | 1.68 | 16.8 | **15** | 8.40 | 84.0 |
| **4** | 2.24 | 22.4 | **16** | 8.96 | 89.6 |
| **5** | 2.80 | 28.0 | **17** | 9.52 | 95.2 |
| **6** | 3.36 | 33.6 | **18** | 10.08 | 100.8 |
| **7** | 3.92 | 39.2 | **19** | 10.64 | 106.4 |
| **8** | 4.48 | 44.8 | **20** | 11.20 | 112.0 |
| **9** | 5.04 | 50.4 | **21** | 11.76 | 117.6 |
| **10** | 5.60 | 56.0 | **22** | 12.32 | 123.2 |
| **11** | 6.16 | 61.6 | **23** | 12.88 | 128.8 |
| **12** | 6.72 | 67.2 | **24** | 13.44 | 134.4 |

**Protocol: Purification of Viral RNA**

Important Note: Change gloves every time hands leave the hood after experiment starts.

1. Take out all samples and let thaw to room temperature.
2. Open n number of QIAamp Mini columns and dump into hood without touching individual tubes (where n = # of extractions).
3. Place filter tubes in 5x16 rack, and label the top of each tube with the number from 1 to n.
4. Shake out 2n 1.75mL microcentrifuge tubes from bag without touching inside of bag (where n = # of extractions).
5. Number the top of each 1.75mL microcentrifuge tube 1 to n for each set.
6. One set of these tubes should also be labeled with the study, extract #, sample ID, and date of extraction on the side of the tube.
7. Bring all thawed samples to hood, vortex, and spin down. Place samples in one 8x12 rack. (See Figure 1 on page 11 for orientation details).
8. Pipet 560 µl of prepared Buffer AVL + carrier RNA into each 1.75 mL microcentrifuge tube.
9. Snap tubes closed.
10. Carefully add 140 µl of plasma/serum sample into their corresponding tube.
    1. Close each lid as sample is added, continually checking gloves for contamination.
       1. If there is any liquid found on the gloves, change gloves and continue (change every 4 samples).
11. Pulse vortex each sample for fifteen seconds.
12. Incubate at room temperature for 10 minutes within hood. Spin down quickly in small centrifuge in the hood.
13. During incubation:
    1. Spray entire centrifuge, inside and out, and surrounding lab space with RNAse, wipe dry. Spray everything again with 70% EtOH, wipe dry. Make sure to dry the inside of wells with Q-tips.
    2. Return samples to freezer.
14. Add 560 µl of 96-100% ethanol to the sample, close lid as ethanol is added
15. Pulse vortex each sample for fifteen seconds. Spin down quickly in small centrifuge in the hood.
16. Add 630 µl of the solution into the QIAamp Mini column without touching the rim. Only one mini column should be open at one time.
17. Centrifuge at 8000 rpm for 1 minute.
18. When taking tubes out of the centrifuge, replace collection tubes with new ones. Discard the filtrate and old collection tubes.
19. Repeat steps 16 through 18.
20. Carefully open the QIAamp Mini column to add 500 µl of Buffer AW1. Only one mini column should be open at one time.
21. Centrifuge at 8000 rpm for 1 minute. When taking tubes out of the centrifuge, replace collection tubes with new ones. Discard the filtrate and old collection tubes.
22. Carefully open the QIAamp Mini column to add 500 µl of Buffer AW2. Only one mini column should be opened at one time.
23. Centrifuge at 14,000 rpm for 3 minutes (our centrifuge only goes up to 13,000 rpm so we run them at 13,000rpm for 3.5 minutes)*Set the centrifuge for 4 minutes and stop after 3.5 minutes
24. Place QIAamp Mini column in labeled microcentrifuge tube with corresponding sample number. Discard the filtrate and old collection tubes
25. Add 50 µl Buffer AVE and let sit for 5 minutes.
26. Centrifuge at 8000 rpm for 1 minute.
27. Keep collection tubes after last centrifuge round.

**Important Note:** If extracts are to be used immediately, First Round RT-PCR reagents should be thawed on lab bench during step 21. Bring samples to lab bench, and immediately proceed to First Round RT-PCR. If extracts are to be amplified at another time, store at -20°C for up to one year.

**Part 2: First Round (1⁰) RT-PCR:**

**Template Preparation**

- Before beginning this step, make sure to make a PCR plate template to keep track of sample locations. For an example of a plate template see below:
- Be sure to maintain the layout in the below plate template and in Figure 1 on page 11 to minimize contamination.

**Master Mix Reagents:**

1. 5X PCR Buffer (300 uL aliquots)
2. 10mM dNTP mix
3. 20uM Forward Primer
4. 20uM Reverse Primer
5. Qiagen One-Step RT-PCR Enzyme Mix
6. RNAsin Plus, RNAse Inhibitor
7. DNase free H20 (1 mL aliquots)

**Cleaning:**

1. Put 3 pipettes (p20, p200, p1000), 2 racks (16x5, 8x12), plate sealer, pen, microcentrifuge in the hood.
2. Spray RNase Away on everything.
3. Clean from right to left including bottom of hood and vortex.
4. Repeat steps 2-3 with 70% ethanol.

**Important Note**: Cleaning should take place before each region of HIV is amplified. For instance, if running gp41 and pol immediately after an extraction, cleaning should be done in between extraction and gp41 first round RT-PCR. Then it should be done again between gp41 first round RT-PCR and pol first round RT-PCR.

**Protocol:**

1. Thaw reagents on lab bench:
   1. 5X, 10 mM dNTP mix, 20uM forward primer, 20uM reverse primer, DNAse free H2O.
   2. Qiagen Enzyme Mix and RNAsin should be kept on a cold block and brought to the hood immediately before added to master mix.
2. Create a PCR program on the thermocycler with the following settings:

| **Thermal Cycler Conditions** | | |
| --- | --- | --- |
|  | **1st ROUND** |  |
| **Pre-PCR** | 50^o^C - 30 minutes |  |
|  | 95^o^C - 15 minutes |  |
| **3-Step Cycling** |  |  |
| denaturation | 94^o^C - 30 seconds |  |
| annealing | 52.5^o^C - 40 seconds | **35 cycles** |
| extension | 72^o^C - 90 seconds |  |
| **Final Extension** | 72^o^C - 10 minutes |  |

1. Calculate the amount of each reagent needed for master mix, by multiplying it by n+2, where n is the number of samples (26x=volumes for a standard plate: 24 samples + 1 negative control = 25 +1 = 26

|  | **RT-PCR GP41 (1ST ROUND)** | | **RT-PCR Pol (1ST ROUND)** | |
| --- | --- | --- | --- | --- |
|  | **1x** | **26x** | **1x** | **26x** |
| **H2O** | 20 uL | 520 uL | 20 uL | 520 uL |
| **5x Buffer** | 10 uL | 260 uL | 10 uL | 260 uL |
| **dNTPs** | 3 uL | 78 uL | 3 uL | 78 uL |
| **primer (1)** | 2 uL | 52 uL | 2 uL | 52 uL |
| **primer (2)** | 2 uL | 52 uL | 2 uL | 52 uL |
| **Enzyme** | 2 uL | 52 uL | 2 uL | 52 uL |
| **Rnase Inhibitor** | 1 uL | 26 uL | 1 uL | 26 uL |
| **MgCl2** | - | - | 2 uL | 52 uL |

|  | ***All primers are diluted to 20uM |
| --- | --- |
|  | **RT-PCR Primers** |
| **Gp41** | gp50 (F), gp41 ® |
| **Pol** | P1F, P1R |

1. Get RT-PCR plate out. Use a **red** plate if doing GP41 and **green** if doing Pol.
2. Label the side of the plate with the study, region, time point, round, initials, date, and plate number.
3. Label the top of the plate with the round number.
4. Vortex all samples and reagents and spin down. Put samples aside.
5. Add 5x Buffer, MgCl₂, dNTP mix, primers to a sterile 5mL tube and vortex.
6. Add enzyme mixture and RNAsin Inhibitor into the master mix. Keep master mix on cold block from now on.
7. Add H₂O then mix gently with pipette tip. DO NOT VORTEX.
8. Load 40 µl of master mix into the wells with an additional well for negative control. When using pipet, go past the first “stop.”

****Switch gloves after every two columns****

**Important Note:** Wells should be loaded down a column as shown by picture below (black indicates a well undergoing PCR reaction). This plate orientation should be maintained until Part 5 of this protocol.


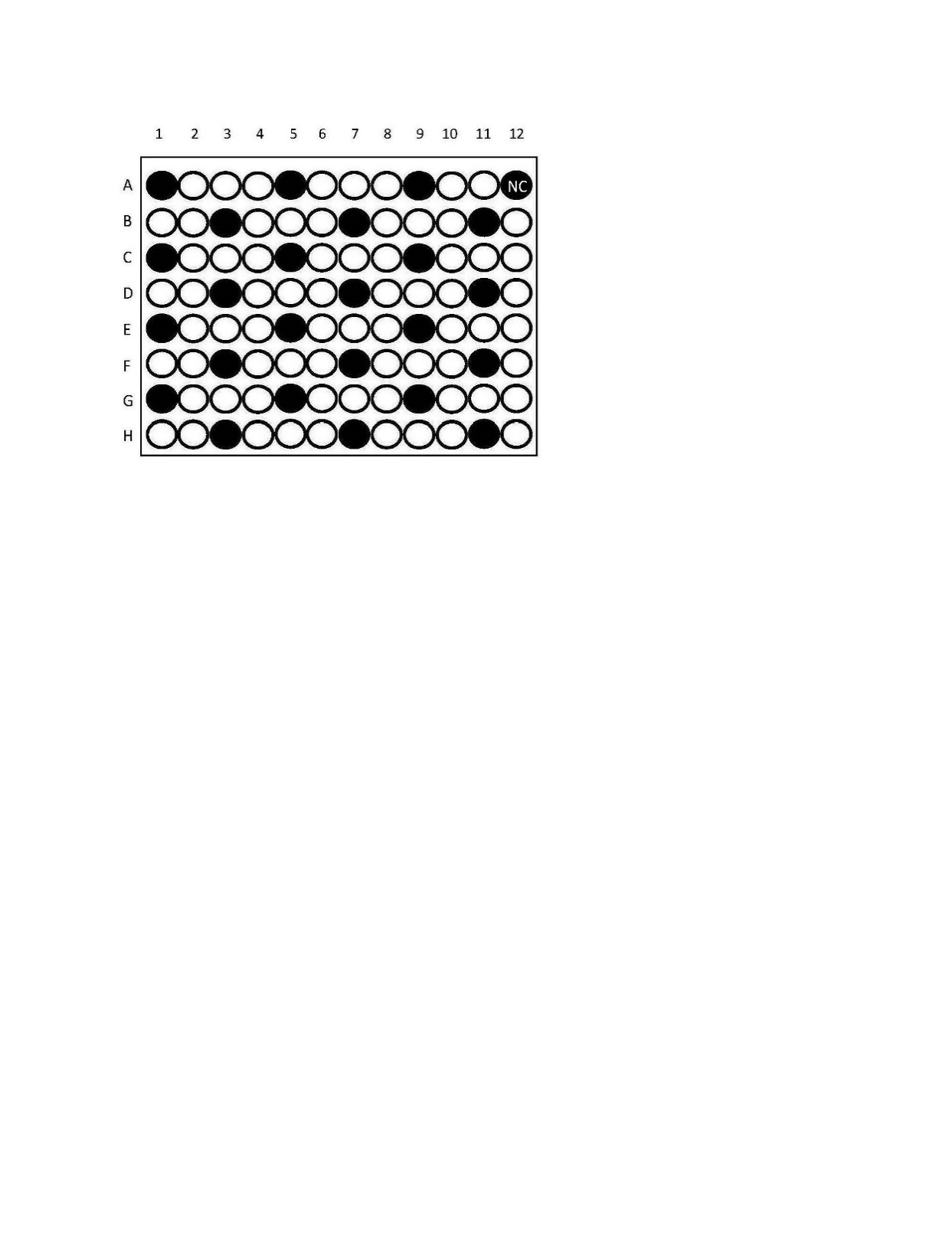


Figure 1

1. Put pop-cap strips over all columns with master mix except first column. **Note:** Only one column should be exposed at a time. After loading a column of extracts, replace strip over column before pulling strip of new column.
   1. Open new bag of strips for each plate/experiment.
2. Load 10 µl extract into specific well. Load down a column.
   1. CAREFULLY! To reduce contamination, refrain from moving pipetter over well that does not contain the sample on the pipetter. Use “Snake method.”
3. Load 10uL H₂O into a negative control well. Cover last column with pop-cap strip.
4. Make sure there are no bubbles in the bottom of the wells. If there are, tap plate lightly to bring bubbles to top of solution. Vortex lightly and centrifuge for 30 seconds at 800 rpm.
5. Place covered plate onto thermocycler and run “MiSeq 1^st^ Round”.
6. Take out after finished and place into -20°C post-amp freezer
   1. Do not leave overnight if not absolutely necessary

**Part 3: Purification of Amplified cDNA Targets**

*Purification should be completed in a separate hood/dead air box.

**Reagent Preparation:**

Once Ampure XP are acquired (Beckman Coulter Agencourt Cat# A63881 – total 60mL), 57 single-use aliquots of 1.04mL should be made in a clean, separate hood/dead air box. Store at 4⁰C.

**Cleaning:**

1. In a separate hood bring all materials to hood, including pipette (p200), 2 PCR plate holders, rack for Ampure XP bead aliquot, magnetic base plate, pen, plate sealer.
2. Spray hood with RNAse Away (do not spray magnetic base plate directly. Spray paper towel first, and wipe down). Wipe dry.
3. Repeat with EtOH. Wipe dry.

**Protocol:**

1. Bring Ampure XP beads to room temperature, and place in hood in rack.
2. Retrieve first round PCR plate from thermocycler or -20⁰C freezer, whichever is applicable, and place on plate holder in hood.
3. Bring a new 96-well plate into the hood and place on the other plate holder.
4. Add 40 µl of resuspended AMPure XP beads to respective wells of the new plate. See plate template on page 4.
5. Transfer 40 µl 1⁰ product to new plate and mix by aspirating/dispensing 10-15 times (homogenous color should be seen).
6. Let sample bind for 10 minutes at room temperature.
7. Place plate on magnetic plate base for 5 minutes (solution should be clear before proceeding).

With plate remaining on the magnetic base:

- 1. Carefully remove 70 µl from each sample well.
     1. Do not disturb magnetic ring!
  2. Add 190 µl of 80% freshly prepared ethanol to each well, then pipette and discard 195µl from each sample well slowly being careful not to disturb the bead pellet.
     1. Be careful to remove all of the EtOH!
  3. Repeat step b for a total of two ethanol washes.
  4. Let plate air-dry for 10 minutes at room temperature.
     1. During this incubation, thaw ready mix for 2^nd^ round master mix.
  5. Add 43 µl reagent grade water to each sample well.

1. Remove plate from magnetic base and snap-cap columns containing reaction, including negative control.
2. Vortex mildly until pelleted beads are in full suspension.
3. Centrifuge the plate at 800 rpm for 30 seconds to ensure ALL liquid is pulled down from the cap.
4. Let sample bind for 5 minutes at room temperature, then place on magnetic base for 5 minutes.
   1. During this incubation, thaw 2^nd^ round primers and clean post-amp lab bench.
5. Pipette 40 µl from each well to a new, labeled 96-well plate. Snap-cap the plate, and immediately place it on a cold plate.
6. Discard the bead purification plate.

**Part 4: Second Round PCR – Addition of Illumina Nextera Sequences to Amplicon-Specific Ends**

**Initial Preparation:** Make labels for each sample (24 per plate). Do not make label for negative control.

**Cleaning:**

1. In pre-amp hood, set all materials inside, including microcentrifuge, P200, P1000 (if applicable), pen, PCR plate holder, rack.
2. Spray hood and equipment with RNAse Away. Wipe dry.
3. Repeat with EtOH. Wipe dry.
4. At post-amp lab bench, set all equipment on the bench, including P2 and plate sealer.
5. Spray bench and equipment with RNAse Away. Wipe Dry.
6. Repeat with EtOH. Wipe Dry.

**Protocol:**

- - - 1. In pre-amp hood, thaw and spin down amplicon specific primers and 2X KAPA HiFi HotStart Ready Mix. Place on cold block.
      2. Create a PCR program with the following cycling conditions:
- 95°C for 3 minutes
- 25 Cycles of:
  - 95°C for 30 seconds
  - 55°C for 30 seconds
  - 72°C for 30 seconds
- 72°C for 5 minutes
- Hold at 4°C

1. Bring 1⁰ plate to post-amp lab bench. Keep it on cold block.
2. Add Master Mix (primers/Ready Mix) as shown below to new labeled plate in pre-amp, and then spin down and bring to post-amp. Set on additional cold block.

| **Component** | **For Single Reaction (1X)** | **For Standard Plate (26x)** |
| --- | --- | --- |
| 2X KAPA HiFi HotStart Ready Mix | 13µl | 338 uL |
| Amplicon Specific Rd1 Forward Primer (1µM) | 5µl | 130 uL |
| Amplicon Specific Rd1 Reverse Primer (1µM) | 5µl | 130 uL |
| Purified amplicon round 1 template | 2µl | - |
| Total Volume | 25µl | - |

1. Add purified amplicon template to corresponding well.
2. Snap-cap the entire plate, vortex to mix, then spin down.
   1. Make sure that there are no bubbles at the bottom of the wells. If there are, spin again.
3. Run 2⁰ amplification program, “MiSeq 2^nd^ Round”.
4. Clean post amp-lab bench as previously described while plate is on the thermocycler.
5. Run gels using 5uL of 2⁰ product.
   1. While loading gels, transfer the rest of the 2⁰ product to a labeled 2mL tube. Store at -20⁰C.
   2. Keep track of how many samples have amplified, and when 96 have accrued continue to the next step “Purification and Normalization of samples from Round 2 PCR.”

**Part 5: Preparation of Third Round PCR Plate—Illumina Indexes**

**Cleaning:**

1. Set up all materials in pre-amp hood.
2. Spray materials and lab bench with RNAse Away, and wipe dry.
3. Spray materials and lab bench with 70% EtOH, and wipe dry.

**Initial Preparation:**

1. Before this step, prepare single-use master mix plates in pre-amp hood. ***See below for a note on how to load this plate***

| **Component** | **For Single Reaction (1X)** |
| --- | --- |
| 2X KAPA HiFi HotStart Ready Mix | 15 µl |
| Nextera XT Index Primer 1 (N7xx) | 5 µl |
| Nextera XT Index Primer 2 (S5xx) | 5 µl |
| Purified, Normalized Round 2 template | 5 µl |
| Total Volume | 30 µl |

- 1. First, load 15ul of 2X KAPA HiFi HotStart Ready Mix to each well of a 96-well plate on plate cooler.
  2. Then, cap columns 2-12 with 8-well domed cap strips. The first column will be loaded first.
  3. Load appropriate amount of each index primer to each well.
     1. Add 5ul of primer N701 to each well in the column. Then add 5ul of each S5xx Index primer (total 8) to corresponding well. *****Take care to change pipet tips so as to not contaminate index primers***** Cap first column. Uncap second column, and repeat with corresponding index primers. See below for a table depicting which primers go in which well.
  4. When finished, store at -20⁰C until ready for use.

**Part 6: Purification and Normalization of Samples from Round 2 PCR**

**Cleaning:**

1. On lab bench separate from post-amp bench used to transfer 1⁰ to 2⁰ plate, set up all materials. These include pipetters (p20, p200), 2 PCR plate holders, 1 rack (5x16). Vortex should already be on bench.
2. Spray materials and lab bench with RNAse Away, and wipe dry.
3. Spray materials and lab bench with 70% EtOH, and wipe dry.

**Protocol:**

1. Add 20 µl of Binding Buffer to each well.
2. Spin down PCR plate *or* tubes.
3. Transfer exactly 20 µl (or all available PCR reaction) to new SequalPrep™ Normalization Plate.
4. Snap-cap the entire plate, vortex to mix, then spin down.
5. Incubate the plate covered at room temperature for 1 hour (overnight incubations possible).
6. Aspirate the liquid from each sample and discard. ***Be sure not to scrape the well sides during aspiration***
7. Add 50 µl Wash Buffer to each well.
8. Snap-cap the entire plate, vortex to mix, then spin down.
9. With a pipette set to 100 µl, carefully aspirate all liquid from each well and discard.
10. Add 20 µl Elution Buffer to each well.
11. Snap-cap the entire plate, vortex vigorously to mix then spin down.
12. Incubate the plate covered at room temperature for 5 minutes.
13. Transfer 5 µl of each sample directly to previously prepared third-round PCR plate on plate cooler (see Part #).
    1. Make sure to only load one column at a time, and to keep all other columns capped while loading a particular column.
    2. Use “snake method” to reduce contamination as much as possible.
14. Proceed to Part 7: Third Round PCR
15. Snap-cap and store normalization plate at -20°C.

**Part 7: Third Round PCR – Addition of Illumina Indexes and Clustering Compatible Ends**

**Cleaning:**

1. In pre-amp hood and on post-amp lab bench, set up all materials.
2. Spray materials and lab bench with RNAse Away, and wipe dry.
3. Spray materials and lab bench with 70% EtOH, and wipe dry.

**Protocol:**

1. Create a PCR program with the following cycling conditions:

- 95°C for 3 minutes
- 8 Cycles of:
  - 95°C for 30 seconds
  - 55°C for 30 seconds
  - 72°C for 30 seconds
- 72°C for 5 minutes
- Hold at 4°C

1. Run **third** round amplification program, “MiSeq 3^rd^ Round”.
2. Transfer 15 µl of each sample to a new, labeled 96-well plate. Snap-cap the plate and prepare the plate for shipment.

**Part 8: MiSeq sample prep and run parameters**

A 96-well plate of individual amplicons is pooled by equal volume amounts (5 ul each) and 150 ul of the resulting pool is purified using an equal amount Ampure XP beads (Beckman Coulter). This purification is modified from the manufacturer’s recommendation, using 400 ul of ethanol for each subsequent wash of the magnetic beads. The beads are then resuspended in 43 ul of EB Buffer (Qiagen) of which 40 ul is aspirated off the pelleted beads in the final step of the manufacturer’s protocol. A portion of the pooled sample is then diluted 1:100,000 in Molecular Biology grade water and quantitated using Illumina Library Quantification Kit (Kapa Biosciences) on a 7900HT real-time PCR instrument (Life Technologies). The quantitated, pooled sample is then sequenced using a MiSeq v3 reagent kit in a paired-end format for 300 cycles in both directions at an 8 pM titration.

**Part 9: Sequence cleaning and analysis pipeline**

The sequencing reads were analyzed and segregated into unique amplicons. Similar amplicons were combined into a single consensus sequence. Consensus sequences that contained >0.02% of the total number of amplicons for that sample were used for all subsequent analyses [[7](#_ENREF_7), [8](#_ENREF_8)]. Phylogenetic trees were created as described above. A representative sequence from each phylogenetically distinct population for every sample on a given MiSeq run (96 samples) was aligned and examined on a neighbor-joining tree for the presence of cross-contamination. Any minor variant that co-localized with a major viral population from another unrelated sample in the run was removed. Detailed method below:

2x300 base reads were trimmed of 5 prime PCR primer sequence using *cutadapt* 1.8 [[9](#_ENREF_9)]. Reads were then trimmed for 3 prime adapter sequence and quality and collapsed into full length amplicons using *AdapterRemoval* [[10](#_ENREF_10)] requiring a minimum of a 15 base overlap of reads. Amplicons containing Ns were filtered out and clustered using *usearch* [[11](#_ENREF_11)] with an identity threshold of 0.98 to generated consensus amplicons. Consensus amplicons were filtered to include only amplicons that contained >0.02% of total raw amplicon number for the given sample. Filtered consensus amplicons were aligned and phylogenetic trees were generated using the neighbor joining method of *clustalw2* [[3](#_ENREF_3)]*.*  Phylogenetic trees were drawn using the ETE *Python* toolkit [[12](#_ENREF_12)].

**Appendix A.1: Primer Sequences for First Round Amplification**

Gp50 (F)

5’ – AAAAATARAACCACTAGGAGTAGCACCCAC – 3’

Gp41

5’ – AACGACAAAGGTGAGTATCCCTGCCTAA – 3’

Pol1F

5’ – ACAGGAGCAGATACAGTATTAGAAG – 3’

Pol1R

5’ – AGTCTTTCCCCATATTACTATGCTTTC – 3’

**Appendix A.2: Primer Sequences for Second Round Amplification**

gp41 Forward

5’- TCGTCGGCAGCGTCAGATGTGTATAAGAGACAGATCTGTTGCAACTCACAGTCTGG -3’

gp41 Reverse

5’- GTCTCGTGGGCTCGGAGATGTGTATAAGAGACAGCTACTATCATTATGAATATTTTTATATACCACAGCC -3’

Pol2 Forward

5’- TCGTCGGCAGCGTCAGATGTGTATAAGAGACAGAATTGGGCCTGAAAATCCATACAATAC -3’

Pol2 Reverse

5’- GTCTCGTGGGCTCGGAGATGTGTATAAGAGACAGGGAGTTCATATCCCATCCAAAGAAATG -3’

*Red bases indicate “template specific” portion of the primer.

**Appendix B: Illumina Indexing adaptors for Third Round PCR Amplification**

| **Catalog Number** | **Item Description** |
| --- | --- |
| FC-131-2001 | Nextera® XT Index Kit v2 Set A (96 Indices, 384 Samples) |
| FC-131-2002 | Nextera® XT Index Kit v2 Set B (96 Indices, 384 Samples) |
| FC-131-2003 | Nextera® XT Index Kit v2 Set C (96 Indices, 384 Samples) |
| FC-131-2004 | Nextera® XT Index Kit v2 Set D (96 Indices, 384 Samples) |

**Appendix C: Additional Reagents and Consumables**

| **Manufacturer** | **Item Number** | **Description** |
| --- | --- | --- |
| Beckman Coulter | A63880 | Agencourt AMPure XP, 5 ml |
| Beckman Coulter | A63881 | Agencourt AMPure XP, 60 ml |
| Life Technologies | A10510-01 | SequalPrep Normalization Plate Kit, 96-well X 10 plates |
| Kapa Biosystems | KK2612 | 2X KAPA HiFi HotStart ReadyMix 250X50ul Rxns |

**New Supplies List:**

- 1 unit of Beckman Coulter Agencourt AMPure XP 60mL. Ref#: A63881
- 1 unit of Life Technologies SequalPrep Normalization Plate Kit, 96-well x 10 plates. Ref#: A10510-01
- 2 units of Kapa Biosystems 2X KAPA HiFi HotStart ReadyMix 250X50ul Rxns. Ref#: KK2612
- Amplicon Primers: All primers should be HPLC purified.
  - gp41 Forward (Scale 1.0)
    - 5’- TCGTCGGCAGCGTCAGATGTGTATAAGAGACAGATCTGTTGCAACTCACAGTCTGG -3
  - gp41 Reverse (Scale 1.0)
    - 5’- GTCTCGTGGGCTCGGAGATGTGTATAAGAGACAGCTACTATCATTATGAATATTTTTATATACC
    - ACAGCC -3’
  - Pol1 Forward (Scale 1.0)
    - 5’- TCGTCGGCAGCGTCAGATGTGTATAAGAGACAGAATTGGGCCTGAAAATCCATACAATAC -3’
  - Pol1 Reverse (Scale 1.0)
    - 5’- GTCTCGTGGGCTCGGAGATGTGTATAAGAGACAGGGAGTTCATATCCCATCCAAAGAAATG -3’
- Illumina Index Primers:
  - Nextera® XT Index Kit v2 Set A (96 Indices, 384 Samples). Ref#: FC-131-2001
  - Nextera® XT Index Kit v2 Set B (96 Indices, 384 Samples). Ref#: FC-131-2002
- 1 unit of Agencourt SPRIPlate 96R - Ring Super Magnet Plate. Ref#: A32782.
- 1 unit of Eppendorf^®^ PCR Cooler, iceless cold storage system for 96 well plates and PCR tubes. Ref#: Z606634-1EA
- 1 unit of Fisher Scientific PCR Workstation/Dead Air Box. 36 in. W, single UV, two fluorescent bulbs. Ref#: S05782

Suggestions for use: The adaptors will come in small microfuge tubes with extra caps. There will be 8 tubes of the (S5xx) adaptors and 12 tubes of the (N7xx) adaptors split by volume into two separate boxes. Upon arrival I would make either strip tube stocks of index combinations or one-time use 96-well arrays of plates then refreeze them. I would not recommend setting up the round 3 PCR plate on-the-fly at the bench. The caps are very small to handle and difficult to spin down, uncap, re-cap while setting up the reactions. It’s very easy to contaminate the adaptors this way.

**IgG antibody isolation from plasma**

Plasma samples were heat inactivated for 1.5 hours at 56°C. From each sample, 500 μL of heat-inactivated plasma was incubated with 450 μL of Protein G Sepharose 4 Fast Flow (GE Healthcare Life Sciences) in 1xPBS (Invitrogen) overnight at 4°C. The Protein G Sepharose bound IgG was washed, 1X PBS, and eluted, elution buffer 0.1 M Glycine of pH 7, then the pH was neutralized with 1 M Tris. Eluted samples were dialyzed and concentrated using the Amicon Ultra-4 Centrifugal Unit with Ultracel-30 membrane (Millipore) and then filter sterilized using Ultrafree-CL Columns, pore size 0.22μm (Millipore). IgG concentrations were obtained using Nanodrop detection at 450 nm for protein.

**Production and titration of HIV-1 pseudoviruses**

The desired *Env* plasmid was combined with pSG3deltaEnv at varied ratios previously determined [[13](#_ENREF_13)] and incubated with the transfection reagent Fugene HD (Roche, Mannheim, Germany) in DMEM for 30 minutes. The mixture was added to a T75 flask of 293T/17 cells and incubated for 48 hours. The supernatants were collected, filtered through a 0.45-micron filter and stored at -80ºC until use. Titration was carried out in TZM-bl cells for 48-72 hours using serial dilutions of 25 µL virus according to the Montifiori protocol [[13](#_ENREF_13)]. Luminescence was detected by adding Bright Glo Reagent (Bright-Glo Luciferase Assay System, Promega, Madison, Wisconsin) to each well for ~2 min and measuring the relative light units (RLU) on a Victor3 Multilabel Counter (PerkinElmer, Waltham Massachusetts). TCID50s were calculated with the TCID50 Excel Macro [[13](#_ENREF_13)].

**TZM-bl neutralization assay**

TZM-bl cells were maintained in Dulbecco's modified Eagle's medium (DMEM with L-glutamine, sodium pyruvate, glucose, and pyridoxine; Gibco-Invitrogen) supplemented with 10% fetal bovine serum (FBS), 2.5% HEPES, and 0.5% gentamicin and passaged twice a week. Heat-inactivated plasma samples from the patients were titrated 1:5-1:1280 in media (above) and mixed with a 200 TCID50 of either primary virus stock or pseudovirus in 96 well plates. If purified total IgG was used instead of heat-inactivated plasma, dilutions were made at 500, 200, 100, 10, 1, and 0.1 µg/mL in DMEM media. After a 1-hour incubation at 37ºC, 10 000 TZM-bl cells in 100 μl of DMEM media containing 10 µg/ml DEAE-dextran were added to each well. The optimal concentration of DEAE for reaching maximum infection was determined for each lot by titration of a 5mg/mL stock solution. The plates were incubated for 48-72 hours at 37ºC, and measured as described above. Neutralization assays with primary virus included 0.2 μl Indinavir per well to limit replication Background luminescence from cells-only wells was subtracted from all RLU values and percent neutralization was calculated by division of the mean RLU for each set of duplicates by the mean RLU in the respective replicates of virus-only control wells, calculations were adjusted with cells only controls and the overall product was multiplied by 100 to obtain the percentage [[13](#_ENREF_13)].

**Production of heterologous virus isolates**

Primary viruses, isolated from Cameroonian patients, were selected from frozen virus stocks. The virus isolates, including tier 2-like 122 (subtype 02_AG), 6491 (subtype G), and 104 (subtype F2) were heterologous to the viruses infecting the study subjects. In addition, tier 1 subtype B viruses SF162 and Bx08 were used. The heterologous viruses were chosen to be able to compare our results with previous studies of discordant superinfection [[14](#_ENREF_14)]. The virus stocks were produced on phytohemagglutinin (PHA) - activated human peripheral blood mononuclear cells (PBMCs), as previously described [[14](#_ENREF_14)]. The cell culture supernatant of the infected cells was tested for p24 concentration using a noncommercial enzyme-linked immunosorbent assay and collected when the p24 concentration reached >100 ng/ml [[15](#_ENREF_15)]. Viruses were titrated like pseudoviruses, as described above.

**Antigen production**

The cyclic V3 peptide (ZM109 sequence) was synthesized by Biomatik (Wilmington, DE). The scaffolded V1V2 (V1V2 ZM109-1FD6) plasmid was obtained from Peter Kwong’s group [[16](#_ENREF_16)] and produced in 293 cells. Briefly, it was cloned into mammalian expression vector pVRC-8400 with a secretion signal sequence at the N-terminus and a His-Tag and a Strep II tag at the C-terminus. The single chain BG505 SOSIP [[17](#_ENREF_17)] was cloned into pVRC-8400 with the same strategy. Both the scaffolded V1V2 and SOSIP scgp140 BG505 were expressed in 293F cells and purified by Ni-NTA beads. The gp120 core (Clade B, JRFL) was cloned into pJW4304 mammalian expression vector with a secretion signal sequence at the N-terminus but without His-Tag; it was expressed in 293F cells and purified by lentil lectin beads.

**ELISA**

Briefly, the antigens (1 µg/ml) were adsorbed onto ELISA plates (Immulon 4HBX; Thermo Fisher, Waltham, MA) overnight at 4^0^C. To reduce non-specific binding, plates were blocked with 3% BSA diluted in PBS/0.05% Tween-20 for 1 hour at room temperature (200 µl per well). Plasma samples were analyzed in1:100 dilutions, IgG samples in 1:5 serial dilutions starting with 500 µg/ml, dissolved in 0.1% Triton-X, for 1.5 hours at 37^0^C. Alkaline phosphatase-conjugated anti-human IgG (1:2000; Southern Biotech, Birmingham, AL) was used as a secondary Ab. P-nitrophenyl phosphate tablets (Thermo Fisher, Waltham, MA) dissolved in diethanolamine (Thermo Fisher, Waltham, MA) were used as the substrate. The optical density was read on a microplate reader (Tecan Sunrise) at 405nm.

**Alignments, epitope and N-glycosylation analysis**

With the help of Box Shade server we highlighted identical amino acids, isofunctional amino acids, and amino acids with different electrochemical properties in comparison to the first time point patient consensus sequence. In order to screen our patient Env consensus sequences for longitudinal changes at key residues for broad neutralizing antibodies, we aligned all patient Env clone sequences and Reference HXB2 using ClustalOmega [[3](#_ENREF_3)]. The prediction of N-linked glycosylation sites was determined using the N-Glycosite tool from the Los Alamos HIV sequence database with sites highlighted in red (<http://www.hiv.lanl.gov/> ). Key N-glycosylation sites and residues that are critical for broad neutralizing antibodies, as well as the site of immune pressure in the RV144 vaccine study, were labeled according to deCamp et al. 2013 [[18](#_ENREF_18)].

**References**

1. Bourlet T, Signori-Schmuck A, Roche L, Icard V, Saoudin H, Trabaud MA, et al. HIV-1 load comparison using four commercial real-time assays. J Clin Microbiol. 2011;49(1):292-7. doi: 10.1128/JCM.01688-10. PubMed PMID: 21068276; PubMed Central PMCID: PMC3020484.

2. Konikoff J, Brookmeyer R, Longosz AF, Cousins MM, Celum C, Buchbinder SP, et al. Performance of a limiting-antigen avidity enzyme immunoassay for cross-sectional estimation of HIV incidence in the United States. PLoS One. 2013;8(12):e82772. doi: 10.1371/journal.pone.0082772. PubMed PMID: 24386116; PubMed Central PMCID: PMC3873916.

3. Thompson JD, Higgins DG, Gibson TJ. CLUSTAL W: improving the sensitivity of progressive multiple sequence alignment through sequence weighting, position-specific gap penalties and weight matrix choice. Nucleic Acids Res. 1994;22(22):4673-80. PubMed PMID: 7984417; PubMed Central PMCID: PMC308517.

4. Tamura K, Dudley J, Nei M, Kumar S. MEGA4: Molecular Evolutionary Genetics Analysis (MEGA) software version 4.0. Mol Biol Evol. 2007;24(8):1596-9. doi: 10.1093/molbev/msm092. PubMed PMID: 17488738.

5. Soulie C, Fofana DB, Boukli N, Sayon S, Lambert-Niclot S, Wirden M, et al. Performance of genotypic algorithms for predicting tropism of HIV-1CRF02_AG subtype. J Clin Virol. 2016;76:51-4. doi: 10.1016/j.jcv.2016.01.010. PubMed PMID: 26826578.

6. Robertson DL, Hahn BH, Sharp PM. Recombination in AIDS viruses. J Mol Evol. 1995;40(3):249-59. PubMed PMID: 7723052.

7. Lehman DA, Wamalwa DC, McCoy CO, Matsen FA, Langat A, Chohan BH, et al. Low-frequency nevirapine resistance at multiple sites may predict treatment failure in infants on nevirapine-based treatment. J Acquir Immune Defic Syndr. 2012;60(3):225-33. doi: 10.1097/QAI.0b013e3182515730. PubMed PMID: 22395670; PubMed Central PMCID: PMC3383885.

8. Redd AD, Collinson-Streng A, Martens C, Ricklefs S, Mullis CE, Manucci J, et al. Identification of HIV superinfection in seroconcordant couples in Rakai, Uganda, by use of next-generation deep sequencing. J Clin Microbiol. 2011;49(8):2859-67. doi: 10.1128/JCM.00804-11. PubMed PMID: 21697329; PubMed Central PMCID: PMC3147722.

9. Martin M. Cutadapt removes adapter sequences from high-throughput sequencing reads. EMBnet journal. 2011;17(1):pp. 10-2.

10. Lindgreen S. AdapterRemoval: easy cleaning of next-generation sequencing reads. BMC Res Notes. 2012;5:337. doi: 10.1186/1756-0500-5-337. PubMed PMID: 22748135; PubMed Central PMCID: PMC3532080.

11. Edgar RC. Search and clustering orders of magnitude faster than BLAST. Bioinformatics. 2010;26(19):2460-1. doi: 10.1093/bioinformatics/btq461. PubMed PMID: 20709691.

12. Huerta-Cepas J, Serra F, Bork P. ETE 3: Reconstruction, Analysis, and Visualization of Phylogenomic Data. Mol Biol Evol. 2016;33(6):1635-8. doi: 10.1093/molbev/msw046. PubMed PMID: 26921390; PubMed Central PMCID: PMC4868116.

13. Li M, Gao F, Mascola JR, Stamatatos L, Polonis VR, Koutsoukos M, et al. Human immunodeficiency virus type 1 env clones from acute and early subtype B infections for standardized assessments of vaccine-elicited neutralizing antibodies. J Virol. 2005;79(16):10108-25. doi: 10.1128/JVI.79.16.10108-10125.2005. PubMed PMID: 16051804; PubMed Central PMCID: PMC1182643.

14. Powell RL, Kinge T, Nyambi PN. Infection by discordant strains of HIV-1 markedly enhances the neutralizing antibody response against heterologous virus. J Virol. 2010;84(18):9415-26. doi: 10.1128/JVI.02732-09. PubMed PMID: 20631143; PubMed Central PMCID: PMC2937625.

15. Laal S, Burda S, Sharpe S, Zolla-Pazner S. A rapid, automated microtiter assay for measuring neutralization of HIV-1. AIDS Res Hum Retroviruses. 1993;9(8):781-5. doi: 10.1089/aid.1993.9.781. PubMed PMID: 8217345.

16. McLellan JS, Pancera M, Carrico C, Gorman J, Julien JP, Khayat R, et al. Structure of HIV-1 gp120 V1/V2 domain with broadly neutralizing antibody PG9. Nature. 2011;480(7377):336-43. doi: 10.1038/nature10696. PubMed PMID: 22113616; PubMed Central PMCID: PMC3406929.

17. Georgiev IS, Joyce MG, Yang Y, Sastry M, Zhang B, Baxa U, et al. Single-Chain Soluble BG505.SOSIP gp140 Trimers as Structural and Antigenic Mimics of Mature Closed HIV-1 Env. J Virol. 2015;89(10):5318-29. doi: 10.1128/JVI.03451-14. PubMed PMID: 25740988; PubMed Central PMCID: PMC4442528.

18. deCamp A, Hraber P, Bailer RT, Seaman MS, Ochsenbauer C, Kappes J, et al. Global panel of HIV-1 Env reference strains for standardized assessments of vaccine-elicited neutralizing antibodies. J Virol. 2014;88(5):2489-507. doi: 10.1128/JVI.02853-13. PubMed PMID: 24352443; PubMed Central PMCID: PMC3958090.
