# Supplementary material for: Contrasting antibody responses to intrasubtype superinfection with CRF02_AG
Source: PLoS One. 2017 Mar 13;12(3):e0173705. doi: 10.1371/journal.pone.0173705 (PMC5348025; doi:10.1371/journal.pone.0173705)
Supplement: S1 Table — (DOCX) [file pone.0173705.s013.docx]

| **Between Time point Mean Genetic Distances in *env*** | | | | | |
| --- | --- | --- | --- | --- | --- |
|  | Time point | 1 | 5 fct | 5 nf |  |
| **NYU6501** | 1 | -- |  |  |  |
|  | 5 fct | 17 | -- |  |  |
|  | 5 nf | 16 | 7.2 | -- |  |
|  | 6 | 16.8 | 2.6 | 7.1 |  |
|  |  |  |  |  |  |
|  | Time point | 1 | 2 | 3 | 4 |
| **NYU6564** | 1 | -- |  |  |  |
|  | 2 | 20.3 | -- |  |  |
|  | 3 | 20.1 | 2.2 | -- |  |
|  | 4 | 20.3 | 2.8 | 2.3 | -- |
|  | 5 | 21.2 | 6.6 | 6.3 | 6.1 |
| **Within Time point Genetic Distances in *env*** | | | | | |
|  | Time point |  |  | Time point |  |
| **NYU6501** | 1 | 0.1 | **NYU6564** | 1 | 0.2 |
|  | 5 fct | 0.3 |  | 2 | 1.9 |
|  | 5 nf | 0.2 |  | 3 | 1.8 |
|  | 6 | 1.9 |  | 4 | 0.2 |
|  |  |  |  | 5 | 0.4 |
| **Between Time point Mean Genetic Distances in *pol*** | | | | | |
| **NYU6501** | Time point | 1 | 5 fct |  |  |
|  | 1 | -- | 2 |  |  |
|  | 4 | 0.7 | 1.7 |  |  |
| **NYU6564** | Time point | 2 | 3 | 4 |  |
|  | 1 | 5.7 | 5.8 | 5.5 |  |
|  | 3 | 1.1 | -- | -- |  |
|  | 4 | 0.8 | 0.7 | -- |  |
| **Between Time point Mean Genetic Distances in *gag*** | | | | | |
| **NYU6501** | Time point | 1 |  |  |  |
|  | 1 | -- |  |  |  |
|  | 5 | 6 |  |  |  |
| **NYU6564** | Time point | 1 |  |  |  |
|  | 1 | -- |  |  |  |
|  | 5 | 5.8 |  |  |  |

**S1 Table. Genetic Distances for HIV-1 genomic regions *env*, *pol*, and *gag*.** Genetic distances in *env*, *pol* and *gag* [11] calculated in MEGA between/within populations, according to phylogenetic trees (see **Fig 2**). For *env*, genetic distances were calculated both between (**top**) and within the time points (**bottom**). For subject NYU6501, time point 5 *env* sequences were distinguished in functional (fct) and non-functional (nf) populations (see **Fig 2**).
