# Supplementary material for: Contrasting antibody responses to intrasubtype superinfection with CRF02_AG
Source: PLoS One. 2017 Mar 13;12(3):e0173705. doi: 10.1371/journal.pone.0173705 (PMC5348025; doi:10.1371/journal.pone.0173705)
Supplement: S2 Table — (PDF) [file pone.0173705.s014.pdf]

	NYU6501			NYU6564		
**EC 50s**	Pre SI 6501-3	Post SI 6501-5	App. Affinity fold diff	Pre SI 6564-1	Post SI 6564-4	App. Affinity fold diff
V1V2	30	3	↑**10**	90	34	↑3
V3	23	12	↑2	31	380	↓**12**
MPER gp41	176	258	≈1	124	144	≈1
gp120 core	4	3	≈1	1	6	↓**6**
SOSIP	44	16	↑3	46	133	↓3

S2 Table. EC50 values of IgG binding to Env antigens. EC50 values and fold changes in apparent affinities after superinfection are listed in the table. Nonlinear regression curves and EC50 values were calculated in GraphPad Prism. In bold are fold changes in apparent affinity greater than 5. Colors of antigens correspond to coloring in main **Fig 4.**
