# Supplementary material for: Contrasting antibody responses to intrasubtype superinfection with CRF02_AG
Source: PLoS One. 2017 Mar 13;12(3):e0173705. doi: 10.1371/journal.pone.0173705 (PMC5348025; doi:10.1371/journal.pone.0173705)
Supplement: S3 Table — (PDF) [file pone.0173705.s015.pdf]

| Sequence | Residues | | | | Nonsynonymous | | | | Isofunctional | | | |
|---|---|---|---|---|---|---|---|---|---|---|---|---|
| | V1V2 | V1 | V2 | V3 | V1V2 | Glycan V2 | V3 | V3 Crown | V1V2 | Glycan V2 | V3 | V3 Crown |
| 6501-1 con | 68 | 30 | 39 | 35 | -- | -- | -- | -- | -- | -- | -- | -- |
| 6501-5 con | 89 | 42 | 48 | 35 | 44 | 2 | 4 | 0 | 8 | 2 | 1 | 1 |
| 6501-6 con | 92 | 45 | 48 | 35 | 43 | 2 | 5 | 0 | 10 | 1 | 1 | 1 |
| Antigen | 66 | 22 | 45 | 35 | 25 | 3 | 6 | 2 | 12 | 4 | 2 | 2 |
| 6564-1 con | 76 | 30 | 47 | 35 | -- | -- | -- | -- | -- | -- | -- | -- |
| 6564-2 con1 | 70 | 31 | 40 | 35 | 22 | 2 | 9 | 3 | 10 | 2 | 1 | 1 |
| 6564-2 con2 | 70 | 31 | 40 | 35 | 21 | 3 | 10 | 4 | 11 | 3 | 1 | 1 |
| 6564-3 con1 | 70 | 31 | 40 | 35 | 21 | 4 | 10 | 4 | 13 | 4 | 1 | 1 |
| 6564-3 con2 | 70 | 31 | 40 | 35 | 22 | 3 | 8 | 3 | 11 | 3 | 1 | 1 |
| 6564-4 con | 66 | 27 | 40 | 35 | 22 | 3 | 8 | 3 | 12 | 3 | 1 | 1 |
| Antigen | 66 | 22 | 45 | 35 | 28 | 4 | 8 | 3 | 8 | 4 | 2 | 1 |

| Sequence | Charge | | | | Tropism | Potential N glycosylation sites | | |
|---|---|---|---|---|---|---|---|---|
| | V1V2 | Glycan V2 | V3 | V3 Crown | | Env | V1V2 | V3 |
| 6501-1 con | 5+ | 2+ | 2+ | 2+ | R5 | 23 | 4 | 1 |
| 6501-5 con | 0 | 0 | 3+ | 2+ | R5 | 28 | 6 | 1 |
| 6501-6 con | 4- | 0 | 4+ | 2+ | R5 | 27 | 6 | 1 |
| Antigen | 0 | 2+ | 4+ | 3+ | R5 | 24* | 5* | 1* |
| 6564-1 con | 1+ | 2+ | 3+ | 2+ | X4 | 27 | 6 | 1 |
| 6564-2 con1 | 2+ | 3+ | 1+ | 1+ | R5 | 25 | 6 | 1 |
| 6564-2 con2 | 2+ | 3+ | 2+ | 2+ | R5 | 26 | 6 | 1 |
| 6564-3 con1 | 2+ | 3+ | 2+ | 2+ | R5 | 26 | 6 | 1 |
| 6564-3 con2 | 2+ | 3+ | 1+ | 1+ | R5 | 26 | 6 | 1 |
| 6564-4 con | 1+ | 3+ | 1+ | 1+ | R5 | 25 | 6 | 1 |
| Antigen | 0 | 2+ | 4+ | 3+ | R5 | 24* | 5* | 1* |

**S3 Table. Comparison of amino acid characteristics of the envelope clones' variable loops.** Number of amino acid residues, nonsynonymous & isofunctional mutations (compared against first time point sequences), potential N-glycosylation sites, tropism, and charges of the V1V2, V1, V2, Glycan V2, V3, V3 crown regions of consensus *Env* sequences from NYU6501 and NYU6564.

Alignments and potential N-glycosylation sites were determined as described in methods. Genotypic prediction of coreceptor tropism was done using Geno2pheno with 5% false positive rate (see methods). V1V2 and V3 antigens as well as HXB2 (indicated with *) were used as Reference strains. Consensus sequences were generated as described in methods; the generation of two consensus sequences was done according to similarity in the highlighter plots in **S2 Fig**. Consensus sequences after SI are filled in with red
